# Single-site ruthenium catalyst supported on zeolite for CO_2_ hydrogenation to methyl formate

**DOI:** 10.1126/sciadv.adu2857

**Published:** 2025-04-16

**Authors:** Roland C. Turnell-Ritson, Lindsey E. K. Frederiksen, Jan Romano-deGea, Barbara Dommen, Darlène S. Dridi, Elio Antonucci, Xunhui Wang, Victor Boureau, Richard Y. Kong, Kyle M. Lancaster, Paul J. Dyson

**Affiliations:** ^1^ Institute of Chemical Sciences and Engineering, École Polytechnique Fédérale de Lausanne (EPFL), 1015 Lausanne, Switzerland.; ^2^ Interdisciplinary Center for Electron Microscopy, École Polytechnique Fédérale de Lausanne (EPFL), 1015 Lausanne, Switzerland.; ^3^ EaStCHEM School of Chemistry, University of Edinburgh, Edinburgh EH9 3FJ, UK.; ^4^ Department of Chemistry and Chemical Biology, Cornell University, Ithaca, NY 14850, USA.

## Abstract

Technologies for the transformation of atmospheric CO_2_ to useful chemicals, such as formic acid (FA), are essential to combatting excessive fossil fuel use and will need to be implemented on large scale. However, hydrogenation of CO_2_ to (base-free) FA is challenging for heterogeneous catalysts, due to the requirement for low temperatures enforced by the entropically unfavorable reaction of gases. By coupling CO_2_ hydrogenation to esterification, methyl formate (MF) can be prepared as a promising alternative platform chemical. Herein, a robust, heterogeneous single-metal-site catalyst was prepared and shown to achieve methanol hydrocarboxylation rates superior to nanoparticle catalysts (up to 18.3 ± 0.6 mmol hour^−1^ $g_{\text{cat}}^{-1}$) while maintaining very high selectivity to MF (≥95%). Characterization reveals isolated, monodisperse Ru-nitrosyl complexes bound to three O-atoms of the zeolite framework, and the robust catalyst formed achieves a cumulative turnover number of more than 3500 over eight cycles. This work pushes the boundaries of supported single-site catalysts in CO_2_ utilization.

## Teaser

A robust, single-site supported Ru complex catalyzes methanol hydrocarboxylation, with excellent selectivity for methyl formate.

## INTRODUCTION

The elimination of petrochemical feedstocks is of utmost importance to reducing excessive CO_2_ emissions and to achieving a sustainable fuels and chemical industry (*1*). Now, carbonylation reactions underpin many of the C–C bond forming processes that produce the bulk of C_2+_ chemicals. Most of the pure CO produced for industry (>500,000 tonnes year^−1^) is used in the carbonylation of methanol (MeOH) to generate acetic acid via the homogeneously catalyzed Cativa and Monsanto processes (*2*, *3*). However, these ubiquitous C_1_ feedstocks (CO and MeOH) are obtained predominantly from fossil sources (gasification of coal gives CO; steam or CO_2_ reformation of light hydrocarbons yields mixtures of CO and H_2_ that are reacted to give MeOH) (*4*).

**Fig. 1.**
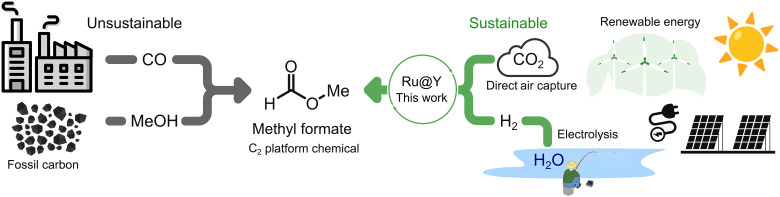
Sustainable platform chemicals. Comparison between the current, fossil-based route to methyl formate (MF), and a sustainable route explored in this work, combining CO_2_ and green hydrogen.

The atmosphere contains an essentially limitless C_1_ reservoir in the form of carbon dioxide. Combining this CO_2_ with green hydrogen (produced from the electrolysis of water) is key to replacing fossil fuels in the chemical industry (Fig. 1). However, efficient routes and catalysts for the partial hydrogenation of CO_2_ to the most versatile C_1_ chemical building blocks (such as formaldehyde) are underdeveloped and not yet commercially competitive, as evidenced by the ongoing scale of fossil fuel–derived processes (*5*–*7*). There is, however, immense interest in designing and developing scalable catalyst systems for CO_2_ utilization (*8*–*10*).

**Fig. 2.**
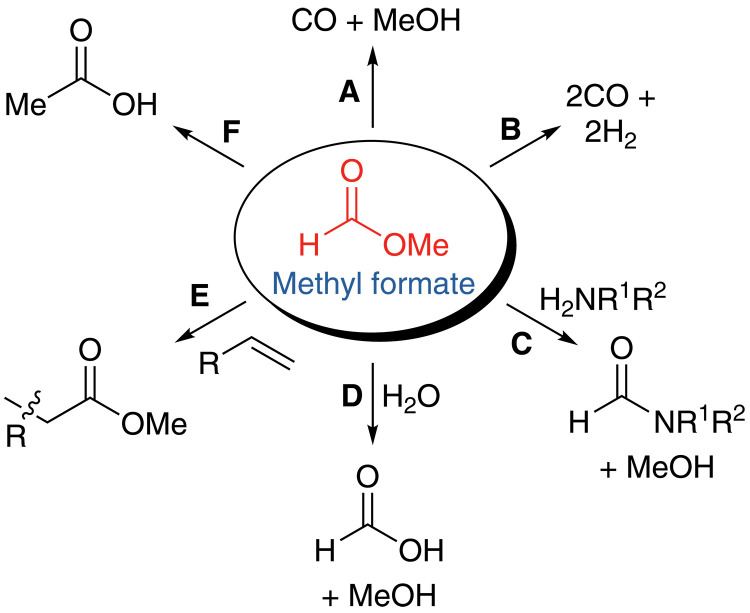
Uses of MF. (**A**) Decarbonylation to CO and MeOH; (**B**) decomposition to syngas; (**C**) aminolysis to amides; (**D**) hydrolysis to FA; (**E**) alkene hydroesterification; (**F**) isomerization to acetic acid.

An alternative target for partial CO_2_ hydrogenation is methyl formate (MF; C_2_H_4_O_2_; Fig. 1). MF is the simplest ester and may be formally considered an isomer of two formaldehyde molecules (CH_2_O). Formaldehyde is an extremely challenging target for CO_2_ hydrogenation, given the ease of reduction of the localized and highly polarized C=O π bond, which makes MF a practical alternative target. At present, more than 90% of the MF produced annually is hydrolyzed directly to give formic acid (FA) (*11*). However, MF itself has been overlooked as a potential platform chemical. As well as yielding both FA and MeOH upon hydrolysis, MF can be reacted with amines to give formamides (*12*), used as a surrogate for the generation of CO or syngas (*13*), and has recently been suggested as an alternative hydrogen carrier which is less toxic than MeOH and less corrosive than FA (Fig. 2) (*14*). MF has also been used directly in the hydroesterification of ethene and higher alkenes (*15*). Showing yet more versatility, MF has been isomerized to acetic acid under CO (*16*–*19*). A recent report revisited the isomerization of MF to acetic acid, using a homogeneous Pd complex in concert with a basic cocatalyst and iodide promoter to perform the isomerization in high yield and good selectivity, without the need for an atmosphere of CO (*20*, *21*). MF is, therefore, an excellent candidate for a platform chemical that can be prepared sustainably from CO_2_ and used to synthesize C_2+_ chemicals.

MF can be prepared from CO_2_ via MeOH hydrocarboxylation (CO_2_ + H_2_ + MeOH → MF + H_2_O), taking place in two steps that together present a substantial thermodynamic challenge (Table 1) (*22*). First, CO_2_ hydrogenation to liquid FA is an exothermic reaction that is highly entropically unfavorable, necessitating low temperatures and high pressures (Eq. 1). Conversely, esterification of FA with MeOH to give MF is both endothermic and entropically favorable and is promoted by high temperatures and lower pressures (Eq. 2). Overall, the hydrocarboxylation is slightly endothermic and somewhat entropically unfavorable, and balancing the two component reactions requires careful control of the reaction conditions (Eq. 3).

**Table 1.** Thermodynamic parameters for MeOH hydrocarboxylation. (1) CO_2_ hydrogenation to FA; (2) FA esterification with MeOH to MF; and (3) overall MeOH hydrocarboxylation to yield MF (*22*).

Eq.	Reaction	Δ*H*^⦵^/kJ mol^−1^	Δ*S*^⦵^/J K^−1^ mol^−1^	Δ*G*^⦵^/kJ mol^−1^
(1)	${\text{CO}}_{2(\text{g})}+{\text{H}}_{2(\text{g})}\to {\text{HCOOH}}_{\left(\text{I}\right)}$	−31.6	−213	+31.8
(2)	${\text{HCOOH}}_{\left(\text{I}\right)}+{\text{CH}}_{3}{\text{OH}}_{\left(\text{I}\right)}\to \text{HCO}\;{\text{OCH}}_{3(\text{I})}+{\text{H}}_{2}{\text{O}}_{\left(\text{I}\right)}$	+35.6	+96.2	+6.9
(3)	${\text{CO}}_{2(\text{g})}+{\text{H}}_{2(\text{g})}+{\text{CH}}_{3}{\text{OH}}_{\left(\text{I}\right)}\to \text{HCO}{\text{OCH}}_{3(\text{I})}+{\text{H}}_{2}{\text{O}}_{\left(\text{I}\right)}$	+4.0	−116	+38.7

Heterogeneous catalysts for MeOH hydrocarboxylation fall into two groups: supported metal nanoparticles (NPs) (*23*–*28*) and polymer- or support-bound metal complexes (*29*, *30*). In general, supported NP catalysts give lower activities [turnover frequencies (TOFs) ≤ 100 hour^−1^] but higher selectivities (>95% selectivity for MF), whereas the supported complexes have higher activities (TOFs ≈ 1000 hour^−1^) but lower selectivities toward MF (50 to 75% selectivity; see table S1 for further details and reported hydrocarboxylation catalysts for comparison purposes). Measurable leaching of the supported complexes has also been detected, particularly in initial cycles, leading to sizeable decreases in activity. At the interface of these two groups is a silica-supported Ru complex, prepared from an acid-catalyzed sol-gel synthesis that traps phosphine-bound Ru complexes in a silica matrix (*31*). The most active of these catalysts recorded a TOF of 115 hour^−1^ over 15 hours under forcing conditions (100°C, 80 bar of H_2_, 135 bar of CO_2_) in MeOH/triethylamine (10:1) as solvent. The Ru-phosphine complex in the silica matrix has similar activity to the homogeneous analog under similar conditions (RuCl_2_[PMe_3_]_4_; TOF = 55 hour^−1^ over 64 hours) (*32*).

Other homogeneous approaches to MF production from CO_2_, H_2_, and MeOH include the use of a Lewis acid additive instead of a base (*33*–*36*) and a two-step CO_2_-to-formate hydrogenation in a biphasic system followed by a reactive distillation to generate and remove MF (*37*). Although these systems typically have the advantage of operating at mild temperatures, the required pressures are still high (>100 bar), and TOFs are comparable to heterogeneous (NP) catalysts (<150 hour^−1^).

With these precedents in mind, we sought to prepare a heterogeneous catalyst for CO_2_ hydrogenation to MF combining the best qualities of all catalyst classes. Consequently, we developed a class of heterogeneous single-metal-site catalysts (*38*, *39*) on the basis of zeolite-supported ruthenium complexes (denoted Ru@Y-*x*) for the hydrocarboxylation of MeOH to give MF. Under optimized conditions, the activity of this catalyst exceeds that of NP catalysts (TOF > 150 hour^−1^) while surpassing the selectivity of polymer-bound complexes (≥95% MF). The catalysts are composed of isolated Ru atoms bound to the zeolite framework, lending stability that is demonstrated in recycling tests, and mechanistic insight into the reaction was also obtained.

## RESULTS

### Catalyst synthesis and characterization

Ru@Y-*x* catalysts were prepared by cation exchange of commercial sodium zeolite Y (NaY) with [Ru(NH_3_)_5_(N_2_)]Cl_2_, synthesized by the reduction of RuCl_3_.*x*H_2_O with aqueous hydrazine (N_2_H_4_) and used in situ (*40*). After filtration, washing, and calcination in air (180°C; 16 hours), the weight loading (*x* wt %) of the Ru@Y-*x* catalyst was determined by inductively coupled plasma mass spectrometry (ICP-MS). Four different weight loadings were prepared, with Ru weight loadings (based on ICP-MS) of 0.1, 1.0, 2.3, and 4.2 wt % (Ru@Y-0.1, Ru@Y-1, Ru@Y-2, and Ru@Y-4, respectively; see Materials and Methods and table S2 for full details). Ru@Y-*x*-NO_3_ catalysts were prepared similarly, with loading of ~1 and 4 wt %, using ruthenium nitrosyl nitrate [Ru(NO)(NO_3_)_3_] as precursor instead of RuCl_3_.*x*H_2_O.

Ultraviolet-visible (UV-Vis) spectroscopy of the zeolite freshly exchanged with [Ru(NH_3_)_5_(N_2_)]Cl_2_ {[Ru(NH_3_)_5_(N_2_)]^2+^@Y-1-RT} shows weak absorptions attributable to oxidized, trimeric [Ru(NH_3_)_5_ORu(NH_3_)_4_ORu(NH_3_)_5_]^6+^ [known as “ruthenium red” (RuRed)] at 375, 550, and 755 nm (fig. S2A) (*41*, *42*). These absorptions grow upon heating to 70°C, as more of the Ru(II) monomer is oxidized to the mixed Ru(III/IV) trimer. This observation was confirmed by exchanging commercial RuRed directly into NaY (RuRed@Y-1-RT; fig. S2B). However, UV-Vis spectra of Ru@Y-*x* (heated to 180°C) no longer show the same features, instead displaying a weak absorption at 450 nm, indicative of the transformation of the RuRed trimer into a different species (fig. S2C). A very similar spectrum is obtained when the exchanged trimer is heated to 180°C overnight (RuRed@Y-1).

The Fourier transform infrared (FTIR) spectra of Ru@Y-*x* give some insight into the nature of this other species (fig. S3). In the FTIR spectrum of Ru@Y-4, a very weak peak at 1875 cm^−1^ is observed, consistent with a linear nitrosyl stretching frequency. The origin of this peak has been previously assigned as [Ru(O_zeolite_)_3_(NH_3_)*_n_*(NO)], where *n* = 1 or 2 (*43*). It is likely that a similar species is present in the Ru@Y-*x* samples of lower loading but is below the detection limit due to its low intensity.

**Fig. 3.**
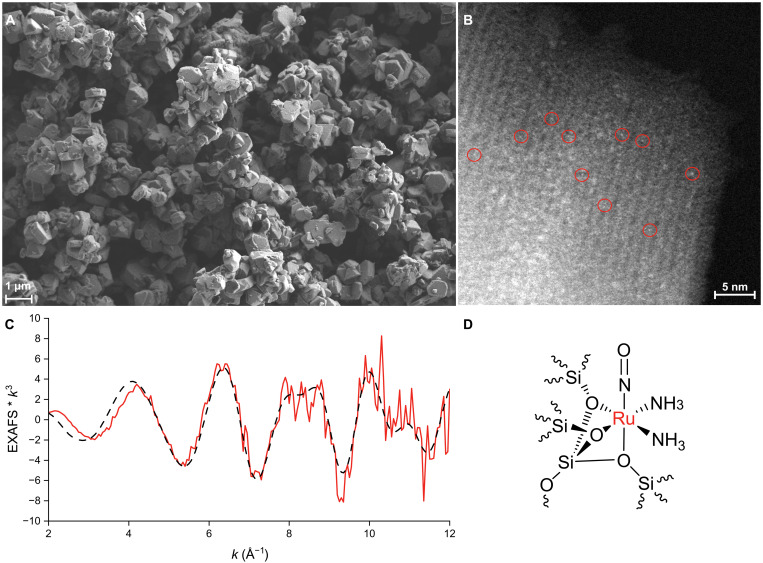
Catalyst characterization. Electron microscopy and extended x-ray absorption fine structure (EXAFS) characterization of Ru@Y-*x* catalysts. (**A**) Scanning electron microscopy (SEM) image depicting the average particle size of Ru@Y-1; (**B**) annular dark-field scanning transmission electron microscopy (ADF-STEM) image of Ru@Y-2, showing a set of crystal planes of zeolite and bright spots assigned to Ru complexes, some of them circled in red; (**C**) EXAFS data for Ru@Y-1 (red solid line) and model (black dashed line) in the region 2 > *k* > 12 Å^−1^; (**D**) tentative structure of the Ru complex bound to the zeolite framework.

To further probe the nature of the Ru species present within the zeolite, electron microscopy techniques were used. Scanning electron microscopy (SEM) and annular dark-field scanning transmission electron microscopy (ADF-STEM) imaging demonstrated the absence of Ru NPs, and showed the average size of the zeolite support particles to be ~400 nm (Fig. 3A and fig. S4). Atomic-resolution ADF-STEM allowed for the observation of Ru atoms on the zeolite crystal support, observed as punctual bright contrasts from the heavier atoms (Fig. 3B and fig. S5). The chemical nature of the heavy atoms was confirmed by energy-dispersive x-ray (EDX) spectroscopy, and the quantified Ru loading was in good agreement with the ICP-MS result (fig. S6 and table S3).

Ru K-edge x-ray absorption spectroscopy (XAS) was used to investigate the physical and electronic structure of the catalytically active species. The Ru K XAS spectrum of Ru@Y-1 was compared to that of a commercial sample of RuRed (fig. S7) and an independently synthesized sample of [Ru(NH_3_)_5_N_2_]I_2_. Both Ru@Y-1 and RuRed are blueshifted relative to [Ru(NH_3_)_5_N_2_]I_2_, suggesting a more oxidized environment for the Ru center. While inspection of the edge position would suggest a blueshift of Ru@Y-1 relative to RuRed, the first derivative maxima are essentially coincident, suggesting similar electron density and, hence, oxidation state, at the Ru centers between the two species.

Extended x-ray absorption fine structure (EXAFS) modeling suggests that the Ru center is six-coordinate and bound to three zeolite O atoms. The first shell is comprised of six O or N scatterers at an approximate distance of 2.06 ± 0.01 Å, which is consistent with previous reports of Ru–O and Ru–N bond lengths and, moreover, similar to the bond lengths of Ru–N bond lengths in [Ru(NH_3_)_5_N_2_]^2+^ as determined by EXAFS (figs. S8 and S9 and tables S4 and S5). A single Si scatterer could also be modeled at a distance of 2.32 ± 0.01 Å from the Ru center, which is consistent with the Si node of the Ru-bound O atoms (Fig. 3C). Interpretation of the second shell of the EXAFS region is more challenging, stemming from uncertainties in the local environment of Ru. Furthermore, numerous multiple-scattering and single-scattering paths are calculated by FEFF (*44*): Three of these paths were chosen and modeled at 3.13 ± 0.02 Å (Si single scatter from the zeolite framework), 3.77 ± 0.04 Å (Si–O multiple scatter), and 3.81 ± 0.05 Å (O single scatter from the zeolite framework and likely H_2_O within the pores). While these data are not conclusive, they are consistent with the proposed model of Ru binding to the zeolite (Fig. 3C).

Powder x-ray diffraction (PXRD) of the parent NaY displayed all the peaks expected for the simulated faujasite structure (fig. S10) (*45*). PXRD plots of the Ru@Y-*x* catalysts showed the same peaks as NaY and no apparent additional features, further demonstrating the intact zeolite structure of the catalysts and suggesting the absence of NPs. Solid-state ^1^H magic angle spinning nuclear magnetic resonance (NMR) spectroscopy of Ru@Y-4 revealed three peaks, at 6.8, 4.3, and 1.1 parts per million (ppm; fig. S11). The most intense of these is the peak at 4.3 ppm (which has a small shoulder at 3.8 ppm), arising from bridging OH groups throughout the zeolite (*46*, *47*). The peak at 6.8 ppm can be assigned to mobile H_2_O molecules or NH_4_^+^ ions, and the small peak at 1.1 ppm is a product of terminal silanol groups. However, the environment of the Ru atoms could not be probed by NMR in the solid state, due to their low loading.

Heating the Ru@Y-*x* catalysts above 180°C generates reduced Ru(0), which has been shown to interact with a number of small molecules (*41*, *48*, *49*). None of the samples, before or after heating to 70° or 180°C, gave any electron paramagnetic resonance (EPR) signal, implying that none of the intermediate or final Ru species are paramagnetic (fig. S12) (*42*, *50*). On the basis of these characterization data, we suggest that the major Ru species present in the zeolite in Ru@Y-*x* catalysts is [Ru(O_zeolite_)_3_(NH_3_)_2_(NO)] (Fig. 3D).

### Catalytic hydrocarboxylation of MeOH

**Fig. 4.**
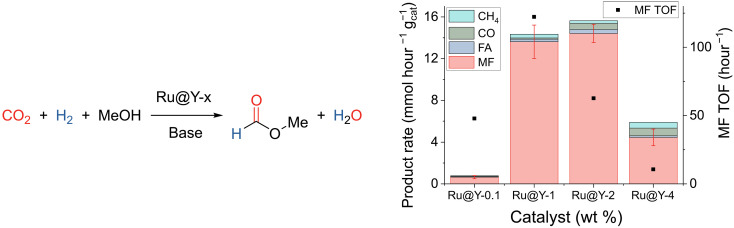
Hydrocarboxylation of MeOH. (**Left**) catalyzed by Ru@Y-*x*, where *x* is the wt % loading of Ru in the catalyst. (**Right**) Variation in the production rate (left axis) and activity for MF (right axis) from the Ru@Y-*x* catalyzed MeOH hydrocarboxylation as a function of the weight loading (*x*). Conditions: Catalyst (25 mg), N-*^n^*Pr-Im (500 mg), MeOH (40 ml), CO_2_ (20 bar), H_2_ (80 bar), 160°C, 16 hours. Average of at least two runs, with SE in MF production depicted.

Ru@Y-0.1, Ru@Y-1, Ru@Y-2, and Ru@Y-4 were tested for MeOH hydrocarboxylation in a batch reactor at 160°C, under an atmosphere of CO_2_:H_2_ (1:4) pressurized to 100 bar, suspended in a MeOH solution containing a weak base (1-*n-*propyl-imidazole; Fig. 4). Gratifyingly, all the catalysts gave the desired MF as the major product. The most active catalyst, i.e., the catalyst with the highest TOF [TOF = mol(MF produced)/mol(Ru)], was found to be Ru@Y-1 (TOF for MF = 122 ± 15 hour^−1^). The rate of MF production relative to the mass of catalyst ($g_{\text{cat}}^{-1}$) was 13.6 ± 1.6 mmol hour^−1^ $g_{\text{cat}}^{-1}$). The Ru@Y-2 catalyst had the same high yield within error (MF production rate = 14.4 ± 0.9 mmol hour^−1^ $g_{\text{cat}}^{-1}$), although a lower specific activity (TOF for MF = 63 ± 4 hour^−1^). The production of CH_4_ may be attributed to Ru NPs, which are effective CO_2_ methanation catalysts, formed under the reductive conditions of the reaction, and observed by STEM imaging of the spent catalyst (fig. S13) (*51*–*54*). It is likely that these Ru NPs are also capable of hydrogenolysis, potentially transforming the MeOH solvent into methane. Higher weight loadings of catalyst would be expected to be more likely to form Ru NPs, due to the increased proximity of Ru sites, and Ru@Y-4 does produce more CH_4_ compared to Ru@Y-1 (6% versus 2%, respectively).

To establish whether the lower activity of the higher weight loading catalyst might be due to the presence of chloride ions from the RuCl_3_.*x*H_2_O precursor, well-known in both heterogeneous (*55*–*57*) and homogeneous catalysis (*58*–*60*), we prepared chloride-free Ru@Y-*x*-NO_3_ catalysts of ~1 and 4 wt % loading using a solution of ruthenium nitrosyl nitrate [Ru(NO)(NO_3_)_3_] instead of RuCl_3_.*x*H_2_O and tested these in the same MeOH hydrocarboxylation reaction (fig. S14). The resulting Ru@Y-1-NO_3_ catalyst has reduced activity compared to the Ru@Y-1 catalyst (TOF = 122 ± 15 versus 87 ± 13 hour^−1^, respectively), but the Ru@Y-4-NO_3_ catalyst has more than double the activity of the Ru@Y-4 catalyst (TOF = 26 ± 3 versus 10.7 ± 1.9 hour^−1^, respectively). However, this value was still substantially reduced compared to the catalysts with weight loading around 1 wt %. Therefore, inhibition by chloride ions accounts for a partial reduction of activity of the high weight loading Ru@Y-*x* catalysts. Furthermore, selectivity toward MF was lower for both Ru@Y-*x*-NO_3_ catalysts compared to their Ru@Y-*x* counterparts.

### Effect of temperature and pressure

**Fig. 5.**
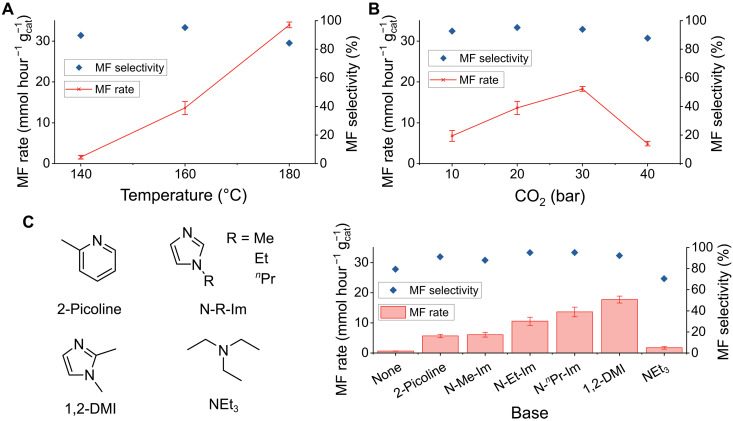
Optimization. Variation in the production rate (left axis) and selectivity toward MF (right axis) from Ru@Y-1 catalyzed MeOH hydrocarboxylation as a function of (**A**) reaction temperature; (**B**) CO_2_ pressure; (**C**) base additive (500 mg). Conditions (unless otherwise stated): Ru@Y-1 (25 mg), N-*^n^*Pr-Im (500 mg), MeOH (40 ml), CO_2_ (20 bar), H_2_ (80 bar), 16 hours. Average of at least two runs, with SE in MF production depicted.

The temperature of the reaction has a substantial effect on the rate of MF production. As the reaction temperature increases from 140° to 160°C, the TOF of MF increases (Fig. 5A). At 180°C, MF production is 34.0 ± 0.7 mmol hour^−1^ $g_{\text{cat}}^{-1}$, corresponding to a TOF of 332 ± 7 hour^−1^. However, there is a notable decrease in the selectivity for the ester (from 95 to 84%). A notable increase in the amount of methane produced was not observed, suggesting that reduction to Ru NPs is not the primary reason for this decrease in selectivity (fig. S15A). Instead, it is likely that decarbonylation of the MF by the base is promoted at higher temperatures, generating more CO. The effect of the base-catalyzed decomposition was explored by subjecting MF to catalytically relevant conditions in the absence of a catalyst. These experiments revealed that, at temperatures of 160°C and above, MF is partially decarbonylated into CO and MeOH (table S6).

The pressure of H_2_ was found to have the expected effect on the rate of production and selectivity toward MF, with both increasing as pressure was increased over the range tested (20 to 80 bar; fig. S15B). Conversely, the effect of CO_2_ was found to be less important, particularly on the selectivity (Fig. 5B and fig. S15C). The optimum MF production rate was obtained at a CO_2_ pressure of 30 bar (TOF for MF = 179 ± 6 hour^−1^; MF production rate = 18.3 ± 0.6 mmol hour^−1^ $g_{\text{cat}}^{-1}$). Excessive CO_2_ pressure (>30 bar) was found to have a sharply negative effect on the rate of MF production. One possible explanation for the decreased activity might be due to changes in the physical properties of the liquid phase under the different reaction conditions. With increasing CO_2_ pressure, the proportion of condensed (both dissolved and supercritical) CO_2_ in the MeOH increases, making the solvent less polar (*61*–*63*). This has a negative effect on the rate of the esterification reaction, thereby decreasing the overall reaction rate. Furthermore, the increased CO_2_ pressure could act as a diluent for the other reactive gas, H_2_, also decreasing the reaction rate (*64*).

### Nature of the base

The nature of the base was varied to find an optimum balance between catalyzing the initial CO_2_ reduction step to FA/formate and permitting the forward esterification reaction. Figure 5C shows the product formation rate for each base, arranged in order of ascending aqueous p*K*_a_ (where *K*_a_ is the acid dissociation constant). Optimum selectivity was observed for 1-alkyl-imidazoles (N-R-Im; R = Me, Et, and *^n^*Pr), where the alkyl chain is at least an ethyl unit (p*K*_a_ = 7.2 to 7.3) (*65*, *66*). Maximum activity was demonstrated for the slightly more basic 1,2-dimethylimidazole (1,2-DMI; p*K*_a_ = 8.21), at the cost of selectivity, with increased CO formation due to the increased rate of MF decarbonylation. The more basic triethylamine (p*K*_a_ = 10.65) (*67*) gave very poor activity and selectivity (see fig. S15D for detailed product breakdown). The optimal performance at intermediate basicity originates from balancing the rates of the two reaction steps (Table 1): the initial base-catalyzed CO_2_ hydrogenation to formate and the subsequent esterification to the methyl ester. If too weak a base is used, then the formate concentration remains too low to permit onward reaction. If it is too high, then the (normally acid-catalyzed) esterification becomes rate limiting. In addition, a strong base can decarbonylate the methyl ester product, resulting in lower perceived activity for MF and much reduced selectivity.

### Nature of the active catalytic species and proposed reaction mechanism

To elucidate the nature of the active catalytic species, a series of control reactions were performed and compared to the standard reaction conditions (Table 2, entry 1). Replacing either of the gaseous reagents with the equivalent pressure of N_2_ caused the rate of MF production to drop to near zero (Table 2, entries 2 and 3), indicating that both the CO_2_ and H_2_ are incorporated into the formyl moiety of the MF product. Small amounts of MF were produced when the reaction was performed without Ru@Y-*x* catalyst (Table 2, entry 4) and, notably, more when the parent zeolite (NaY) was added (Table 2, entry 5). Note that protonated zeolites (e.g., HY) are known to catalyze esterification reactions (*68*–*70*), and NaY has also been demonstrated to be catalytically active for the esterification of oleic acid to methyl oleate (*71*).

**Table 2.** Control experiments. Comparison of the MF productivity of Ru@Y-1 under various atmospheres, and against homogeneous Ru catalysts.*

Entry	Catalyst	CO_2_/bar	H_2_/bar	N_2_/bar	MF production rate/μmol hour^−1^	MF selectivity/%
1	Ru@Y-1	20	80	0	340 ± 40^†^	95
2	Ru@Y-1	0	80	20	1.4	16
3	Ru@Y-1	20	0	80	3.0	82
4	None	20	80	0	22	87
5	NaY	20	80	0	57	89
6	Supernatant	20	80	0	14 ± 2^†^	88
7	[Ru(NH_3_)_5_(N_2_)]I_2_	20	80	0	270 ± 50^†^	95
8	Ruthenium red chloride	20	80	0	300 ± 20^†^	95
9	Ru@3ÅMS-0.1	20	80	0	361 ± 15^†‡^	39
10	Ru@ZSM-5-0.5	20	80	0	241 ± 4^†‡^	72
11	Ru@Y-1	1	80	3^§^	10.3 ± 1.3^†^	36

*Conditions unless stated otherwise: 2.5 μmol of Ru, 500 mg of N-*^n^*Pr-Im, 40 ml of MeOH, 20 bar of CO_2_, 80 bar of H_2_, 160°C, 16 hours.

†SEs calculated from an average of at least two runs.

‡Normalized to 2.5 μmol of Ru (25 mg of catalyst used).

§2 bar of N_2_, 1 bar of air.

A filtered reaction solution (from Table 2, entry 1) was tested by ICP-MS for leaching of Ru and found to contain 64-ppm Ru, equating to a leaching rate of 2.6%. This supernatant had all the volatiles removed in vacuo and the residue redissolved in fresh MeOH, before resubjecting it to the standard reaction conditions (Table 2, entry 6). MF production was in line with the catalyst-free control (Table 2, entry 4), indicating that leached Ru is not catalytically active.

The homogeneous complexes, monomeric [Ru(NH_3_)_5_(N_2_)]I_2_ and trimeric RuRed chloride, were evaluated as catalysts in the hydrocarboxylation of MeOH (at the same Ru concentration present in the Ru@Y-1 reactions) and were found to have similar activities to Ru@Y-1, within error (Table 2, entries 7 and 8). This similarity is despite the expectation that attaching the active Ru species to the zeolite framework reduces translational and rotational flexibility, thereby decreasing the rate of steps that require topological rearrangements of the ligands around the Ru center. In all three cases, the selectivity was found to be 95%, another indication that a similar hydrogenation mechanism may be performed by each Ru species. Ex situ characterization techniques were used to explore the changes to the catalyst during the reaction. A PXRD pattern of used Ru@Y-1 reveals no new peaks but does show that every peak is broadened compared to that of fresh catalyst (fig. S16). This broadening can be explained by a reduction in size of the zeolite crystallites, caused by the mechanical stirring.

To examine the role of the zeolite support, the synthesis method for Ru@Y-1 was applied to two different materials: Linde Type A 3-Å molecular sieves (3ÅMS) and zeolite ZSM-5. This yielded Ru@3ÅMS and Ru@ZSM-5, respectively. In both cases, exchange of the Ru precursor onto the zeolite was found to be incomplete, with ICP-MS returning Ru loadings of 0.1 and 0.5 wt %, respectively, despite the targeted 1.0 wt %. When applied to the hydrocarboxylation of MeOH, it was found that Ru@3ÅMS-0.1 had similar (normalized) activity (i.e., TOF; see fig. S17) for MF but poor selectivity at only 39% (Table 2, entry 9). In the case of the ZSM-5, the activity was less than Ru@Y-1, and, again, the selectivity was decreased to only 72% (Table 2, entry 10). In both cases, the main reaction by-product was methane, suggesting the formation of Ru NPs, either in synthesis or during the reaction. These results imply that the structure of zeolite-Y assists in the formation of monodisperse Ru species, which are stable under the reaction conditions and selective for CO_2_ hydrogenation to formate.

The Ru@Y-1 catalyst was also tested for CO_2_ conversion using simulated flue gas (Table 2, entry 11), to assess its applicability to an impure CO_2_ stream. The flue gas was simulated by combining CO_2_ (1 bar), air (1 bar), and N_2_ (2 bar) to give a mixture of CO_2_, O_2_, and N_2_ in 25:5:70 ratio. Under these conditions, the catalyst activity was greatly decreased, in line with the 20-fold decrease in CO_2_ pressure. Unfortunately, selectivity was also affected, likely due to the presence of O_2_ damaging the catalyst active sites. For real-world application, therefore, further testing of gas purity would be required to determine what impurities the catalyst can tolerate.

FTIR spectra of used Ru@Y-1 and Ru@Y-4 revealed new stretches in the region of 2900 to 3200 cm^−1^ (attributable to C–H stretches of adsorbed methoxide species) (*72*), and new peaks in the region of 1200 to 1950 cm^−1^, which are not readily assigned (fig. S18). The spectrum of used Ru@Y-4 reveals the absence of the NO stretch identified in the fresh catalyst, implying that the NO ligand is not retained during catalysis.

Performing the reaction under an atmosphere of N_2_ in place of CO_2_ (as in Table 2, entry 2) replicates many of the peaks in the region of 1400 to 1600 cm^−1^, suggesting that they may be attributed to MeOH or NH_3_ bending modes (fig. S19). However, a small additional peak is observed in the spectrum of Ru@Y-1 used under CO_2_ at 1315 cm^−1^, which is absent from the spectrum of Ru@Y-1 used under N_2_. Two simplified models of a zeolite-bound Ru(II)-formate complex, [(HOSi(OR)_3_)Ru(OOCH)(NH_3_)*_n_*]^+^ [R = Si(OH)_3_], were constructed, where the OOCH moiety is bound in either a monodentate (*n* = 2) or bidentate (*n* = 1) fashion (figs. S20 and S21). Their geometry was optimized using density functional theory (DFT) at the M062X level using the def2-TZVPP basis set. Using these optimized geometries, simulated infrared (IR) spectra were generated (fig. S22). A peak is present in both simulated spectra at ~1320 cm^−1^, attributable to a symmetric ν(COO) stretch (*73*). For the monodentate complex, the asymmetric ν(COO) stretch was simulated at 1740 cm^−1^, whereas, for the bidentate model complex, the asymmetric ν(COO) stretching mode was simulated at 1605 cm^−1^. Due to the absence of peaks at ~1700 cm^−1^ and as any peaks around 1650 cm^−1^ would overlap with the strong zeolite-bound H_2_O signal, the peak at 1315 cm^−1^ in the spectrum of used Ru@Y-1 was tentatively assigned to a bidentate Ru-formate intermediate.

**Fig. 6.**
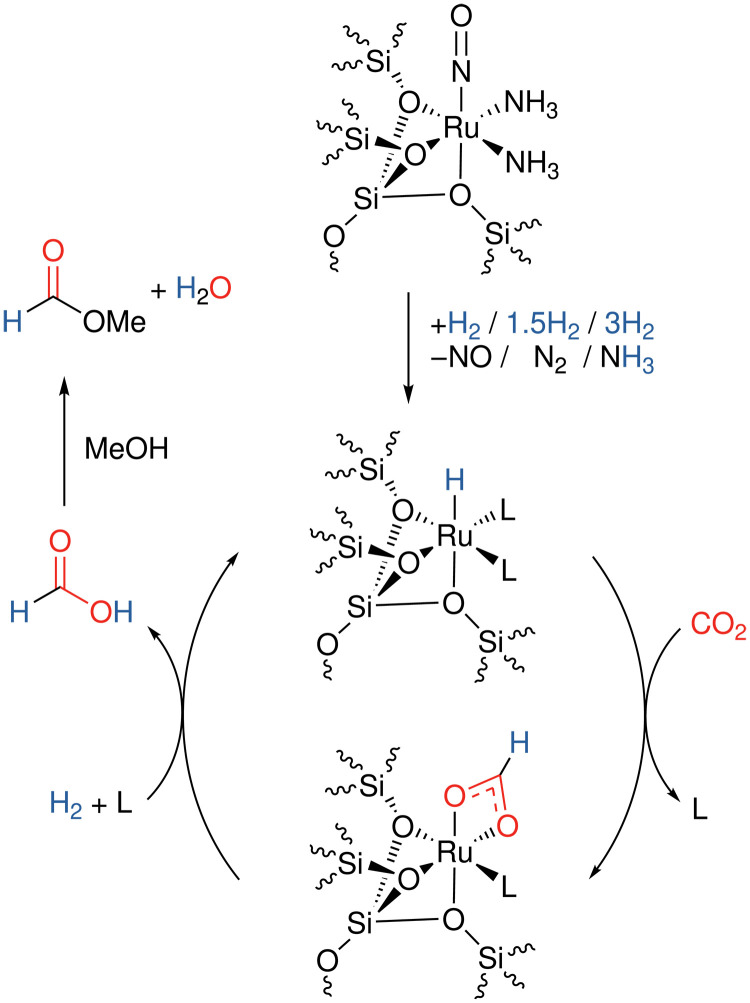
Proposed mechanistic cycle for Ru@Y-*x* catalyzed CO_2_ hydrogenation, and subsequent esterification. L = NH_3_, H_2_O, or MeOH.

On the basis of these observations, we propose the catalytic cycle depicted in Fig. 6. As the NO ligand is not observed after the reaction, we suggest that the precatalytic species, [Ru(O_zeolite_)_3_(NH_3_)_2_(NO)], loses an NO ligand upon exposure to hydrogen, either by displacement or hydrogenation of NO to N_2_ or NH_3_, as observed with other heterogeneous Ru catalysts (*74*). Subsequently, CO_2_ can insert into the Ru–H bond, giving a bidentate Ru-formate, with concomitant dissociation of a ligand from the Ru center. Reaction of the Ru-OOCH with H_2_ generates the FA product and regenerates the Ru–H catalyst. The FA then undergoes esterification with the MeOH solvent. This mechanism is consistent with previous studies on catalytic CO_2_ hydrogenation using homogeneous Ru complexes (*75*). From this ex situ IR data, the catalyst resting state appears, therefore, to consist of a κ^2^-Ru-OOCH species, and not a Ru–H species. However, due to the oxidized nature of the Ru (a similar 3+/4+ oxidation state to RuRed, from the XAS data, vide supra), the Ru centers are likely more oxophilic than typical Ru(II) hydrogenation catalysts, and the corresponding Ru–H bond is destabilized.

### Recycling studies

**Fig. 7.**
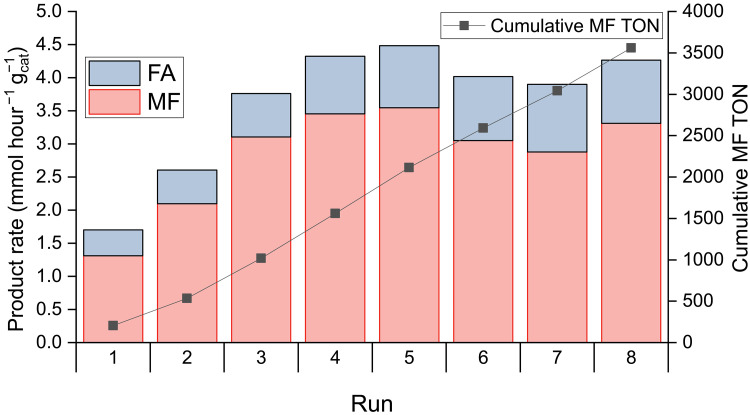
Recycling study. Productivity toward liquid products of Ru@Y-1 catalyzed MeOH hydrocarboxylation (left axis) and cumulative MF turnover number (TON) for eight reaction cycles (right axis). Conditions for each run: Ru@Y-1 (12.5 mg), N-*^n^*Pr-Im (250 mg), MeOH (20 ml), CO_2_ (20 bar), H_2_ (80 bar), 160°C, 16 hours. Average of two runs.

Recycling of the catalyst was performed under conditions facilitating reuse of the catalyst (Fig. 7 and fig. S23). Due to a reduction in both headspace (20 ml instead of 35 ml) and solvent volume (20 ml instead of 40 ml), this led to a reduction in the activity of the catalyst, and selectivity toward MF was also decreased. However, MF remained the major product in every run. Activity increases over the first four cycles, indicative of a long induction period. Previous studies on Ru complexes trapped inside a silica matrix also noted increased turnovers after the first catalytic run (*30*), attributed to the formation of colorless Ru-hydrides during catalysis, which were posited to be the active catalyst for CO_2_ hydrogenation. The initially pink Ru@Y-*x* also loses its color after reaction, although FTIR spectroscopy of the used catalyst did not reveal any Ru–H stretches (fig. S17). This finding contrasts with polymer-bound Ru catalysts, which display strong Ru-CO and Ru–H stretching peaks after use (*28*, *29*). Alternatively, the increasing surface area of the catalyst, as the zeolite particles are broken into smaller pieces, could explain the increasing activity. From run 5 onward, the activity trends slightly downward, suggesting that catalyst deactivation is very slow. These recycling results are indicative of a robust catalyst, with a cumulative turnover number of more than 3500 obtained over eight cycles.

## DISCUSSION

We have prepared an active class of heterogeneous catalysts, Ru@Y-*x*, for the hydrocarboxylation of MeOH to MF, which display excellent selectivity. Comprehensive characterization revealed the catalysts to consist of isolated, monodisperse Ru-NO complexes bound to the zeolite framework via three zeolite oxygen atoms. The monodisperse nature of the Ru sites likely contributes to the high observed selectivity for MF (up to 95%). We have investigated the effect of the catalyst preparation and reaction conditions on the activity and selectivity of the catalyst, with TOF for MF reaching 179 ± 6 hour^−1^. We also demonstrated the recyclability of the catalyst, with excellent cumulative activity for MF production. Such robustness suggests that, should the present process be sufficiently scalable, it could complement an e-MeOH plant, in which all of the raw materials (CO_2_, H_2_, and MeOH) are produced on-site with the aid of renewable energy (wind and solar) (*6*, *7*). Control reactions and ex situ catalyst analysis give some insight into the catalytic cycle, suggesting the formation of catalytically active Ru–H and Ru-OOCH intermediates. We hope that this work inspires the further development of similar molecular-type single-metal-site catalysts, which bridge the gap between homogeneous catalysis and industrial application, for CO_2_ utilization.

## MATERIALS AND METHODS

### Materials

RuCl_3_.*x*H_2_O (99%; Precious Metals Online), Ru(NO)(NO_3_)_3_ in dilute nitric acid (Ru, 1.5 wt %; Sigma-Aldrich), N_2_H_4_ monohydrate (98%; Sigma-Aldrich), [(NH_3_)_5_RuORu(NH_3_)_4_ORu(NH_3_)_5_]Cl_6_ (RuRed; 90%; Sigma-Aldrich), *N*-*n*-propylimidazole (N-*^n^*Pr-Im; 97%; Combi-Blocks), *N*-ethylimidazole (N-Et-Im; 98%; abcr), *N*-methylimidazole (N-Me-Im; 99%; Fluorochem), 1,2-DMI (98%; Alfa Aesar), triethylamine (Et_3_N; 99.5%; Thermo Fisher Scientific), 2-methylpyridine (2-picoline; 98%; Sigma-Aldrich), MeOH (99.9%; Thermo Fisher Scientific), MF (99%; Sigma-Aldrich), *d*_6_–dimethyl sulfoxide (*d*_6_-DMSO; 99.8% deuterium; Eurisotop), and *para-*xylene (99%+; Sigma-Aldrich) were purchased from commercial suppliers and used without further purification. [Ru(NH_3_)_5_N_2_]I_2_ was prepared via the literature route (*40*). Sodium zeolite-Y (NaY; SiO_2_:Al_2_O_3_, 5.1:1; surface area 900 m^2^ g^−1^) and Linde Type A 3ÅMS (SiO_2_:Al_2_O_3_, 2:1) was purchased from abcr, and zeolite type ZSM-5 was purchased from Acros Organics. All three were calcined at 250°C before use. CO_2_ (99.998%), H_2_ (99.999%), and N_2_ (99.9999%) were purchased from Carbagas.

### Catalyst synthesis

To prepare Ru@Y-1, N_2_H_4_ monohydrate (260 μl) was added dropwise to RuCl_3_.*x*H_2_O (259 mg, 38.2 mmol) dissolved in Milli-Q water (5 ml) and left to stir overnight under an atmosphere of N_2_. Subsequently, sodium zeolite-Y (NaY; 10 g) was added, the suspension diluted with Milli-Q water (95 ml) and, again, stirred overnight (under an atmosphere of N_2_). The solid was removed by filtration and washed several times with distilled water. The solid was then heated to 180°C for 16 hours to give Ru@Y-1. The weight loading of the catalyst was changed by varying the amount of RuCl_3_.*x*H_2_O and N_2_H_4_ monohydrate as appropriate and denoted as Ru@Y-*x* (*x* = wt % of Ru in the sample, as measured by ICP-MS). For the Ru@Y-*x*-NO_3_ catalysts, Ru(NO)(NO_3_)_3_ in dilute nitric acid was used in place of RuCl_3_.*x*H_2_O. For RuRed@Y-1, [Ru(NH_3_)_5_ORu(NH_3_)_4_ORu-(NH_3_)_5_]Cl_6_ (RuRed) was used in place of RuCl_3_.*x*H_2_O, and hydrazine was omitted. For Ru@3ÅMS-0.1, 3-Å molecular sieve powder used in place of NaY. For Ru@ZSM-5-0.5, ZSM-5 was used in place of NaY.

### Catalyst testing

Catalytic tests were performed by loading the Ru@Y-*x* or Ru@Y-*x*-NO_3_ catalyst (25 mg), MeOH (40 ml), and a base (500 mg) into a stainless-steel Parr reactor (75 ml) along with a glass-coated magnetic stirrer bar, followed by degassing the solution three times with CO_2_ (20 bar) and then pressurizing the reactor sequentially with CO_2_ and H_2_ to the desired pressures. The reactor was then placed in a preheated mantle set to the desired temperature over a magnetic stirrer plate set to 500 rpm, and the reaction quenched by placing the hot reactor in cold water and then ice. Analysis of the gaseous products, collected in a Tedlar gas sampling bag, was performed by gas-injection gas chromatography (GC) equipped with a PoraPLOT Q column and coupled with both thermal conductivity detector (TCD) and flame ionization detector (FID; for a representative example, see fig. S23). Liquid products were analyzed by ^1^H NMR spectroscopy (Bruker Avance III HD-400), using *para*-xylene as internal standard. After venting the reactor, *para*-xylene (~500 mg) was weighed into the autoclave and then stirred until thoroughly mixed with the reaction mixture. The mixture (10 ml) was filtered through a Teflon syringe filter (0.45 μm), and 0.5 ml of the filtered solution was added to an NMR tube along with 0.2 ml of *d*_6_-DMSO (for a representative example, see fig. S24).

### Base-induced decarbonylation of MF

MF (480 mg; 0.20 M), N-*^n^*Pr-Im (500 mg; 0.11 M), and MeOH (40 ml) were added into a stainless-steel Parr reactor (75 ml) with a glass-coated magnetic stirrer bar, followed by degassing the solution three times with CO_2_ (20 bar) and then pressurizing the reactor sequentially with CO_2_ (20 bar) and H_2_ (80 bar). The reactor was subsequently placed in a preheated mantle set to the desired temperature over a magnetic stirrer plate set to 500 rpm, and the reaction quenched by placing the hot reactor in cold water and then ice. Analysis of the gaseous and liquid products was performed as above (table S6).

### Recycling tests

Recycling tests were performed similarly to the catalytic tests, but with some changes to enable recovery of the catalyst. Ru@Y-1 (12.5 mg), MeOH (20 ml), and N-*^n^*Pr-Im (750 mg) were loaded into a stainless-steel Parr reactor with a removable glass sleeve (40 ml) along with a glass-coated magnetic stirrer bar, followed by degassing the solution three times with CO_2_ (20 bar) and then pressurizing the reactor sequentially with CO_2_ (20 bar) and H_2_ (80 bar). The reactor was then placed in a preheated mantle set to the 160°C over a magnetic stirrer plate set to 500 rpm, and the reaction quenched after 16 hours by placing the hot reactor in cold water and then ice. Analysis of the gaseous products, collected in a Tedlar gas sampling bag, was performed by gas-injection GC equipped with a PoraPLOT Q column and coupled with both TCD and FID. Liquid products were analyzed by ^1^H NMR spectroscopy (Bruker Avance III HD-400), using *para*-xylene as internal standard. After venting the reactor, *para*-xylene (~500 mg) was weighed into the glass sleeve and then stirred until thoroughly mixed with the reaction mixture. The solution (0.2 ml) was added to an NMR tube along with 0.4 ml of *d*_4_-MeOH, which was returned to the glass sleeve after spectra were collected. All volatiles from the reaction were then removed in vacuo (70°C, 10 mbar), leaving only the catalyst suspended in the base and the stirrer bar (complete removal of *para*-xylene, MeOH, and formates was confirmed in an independent test). To prepare the next run, the glass sleeve was returned to the autoclave, charged with MeOH (20 ml), and repressurized as above.

### Leaching tests

A filtered reaction solution was tested by ICP-MS for leaching of Ru and found to contain 64-ppm Ru. This equates to a leaching rate of 2.6%. It is likely that this Ru originates from metal atoms that have not been fixed to the zeolite structure, which would result in less leaching on subsequent cycles.

### Instruments

Solution-phase ^1^H NMR spectra were recorded on a Bruker Avance III HD-400 instrument. GC was performed on an Agilent 7890B GC System equipped with a PoraPLOT Q column and FID. PXRD patterns were acquired using a Bruker D8 Discover Vario. FTIR spectra were collected using a PerkinElmer Spectrum Two FT-IR spectrometer. UV-Vis spectra were collected using a PerkinElmer Lambda 950 UV/Vis/NIR Spectrophotometer.

### Electron microscopy

SEM images were recorded on a Zeiss GeminiSEM 300 instrument equipped with a standard Everhart-Thornley secondary electron detector and a Gemini II column with an InLens secondary-electron detector. The instrument was operated in high vacuum mode. High-angle ADF-STEM (HAADF-STEM) images were recorded on a FEI (now Thermo Fisher Scientific) Talos F200S instrument operated at 200-keV beam energy and equipped with a Fischione HAADF detector. Atomic-resolution ADF-STEM images and EDX spectra were collected on a FEI Titan Themis instrument using a 300-keV beam energy. The microscope is equipped with a high-brightness Schottky emission gun (X-FEG) and a probe aberration corrector (CEOS D-COR), and data were collected using a Fischione HAADF detector for STEM imaging and four silicon drift detectors (SDD Super-X) for EDX. Data were collected with an electron probe of 25 pA and a convergence angle of 20 mrad. ADF-STEM used a 26- to 155-mrad collection angle and 1-μs dwell time. EDX spectra were analyzed with Velox software, using the Cliff-Lorimer method from the K-lines of O, Na, Al, Si elements, and L-line of Ru; the Brown-Powell ionization cross-sections were used.

### Inductively coupled plasma mass spectrometry

Samples were weighed and submitted to acidic digestion with 4 ml of aqua regia consisting of concentrated acids HNO_3_ (69%, ROTIPURAN Supra, Roth) and HCl (35%, ROTIPURAN Supra, Roth) freshly mixed in 1:3 ratio. Digestion was performed using a microwave oven (ETHOS.lab, MLS MWS Mikrowelle GmbH) set to heat to 210°C over 15 min and maintained at 210°C for 25 min. After digestion, the samples were diluted 300 times with 2% HNO_3_ solution, and the metals of interest were quantified by ICP-MS using kinetic energy discrimination mode with He as the collision gas on a NexIon 350 D ICP-MS instrument (PerkinElmer). Yttrium was added as an internal standard at a concentration of 2 parts per billion (ppb) to the solution, and metals quantitation was performed using an external calibration curve with standards in the 0.05- to 50-ppb range. All measurements were performed in triplicate.

### X-ray absorption spectroscopy

XAS measurements were conducted at the BM31/Swiss Norwegian Beamlines at the European Synchrotron Radiation Facility (ESRF) in Grenoble, France. The polychromatic beam from the two-pole wiggler was monochromatized using a Si(111) LN_2_-cooled monochromator, and the parallel beam was defined by the use of slits to a size of 3 mm (horizontal) by 300 μm (vertical).

Data were acquired from pressed pellets (1.3 cm^2^) properly diluted with an inert substance (i.e., cellulose) and an appropriate thickness (typically of two to three absorption lengths) at the Ru K-edge in transmission mode, using two ionization chambers placed before and after the pellet and optimized to 20% absorption in the monitor (*I*_0_) and 80% absorption in the transmitted chamber (*I*_1_). An absorption step around 1 was always obtained. For the absolute energy calibration, a metal foil was measured simultaneously between the second and third ionization chambers. All 3-cm-long ionization chambers were filled with inert gases and optimized to the numbers mentioned above. Typically, a scan was performed using around 0.5-eV step size and 100-ms integration time per point resulting in a total scan time of ~4 min (minimum of 20 repetitions for improved statistics, better signal to noise ratio, etc.). The resulting spectra were energy calibrated, background corrected, and merged using the Larch software package (version 0.9.79) (*76*). The energy of the incident beam was calibrated to the first inflection point of Ru foil set at 22,117 eV. Spectra were normalized using the MBACK algorithm, with the pre-edge defined as below 22,080 eV.

EXAFS were modeled using the Larch software package (*44*, *76*, *77*). Spectra were fit in *R*-space with a *k*-weighting of 3. Background subtraction was performed using an *R*_bkg_ = 1.0 and a *k*-weight of 3 between *k* = 2 and 16. *S*_0_^2^ was fixed at 0.8.

### EPR spectroscopy

EPR spectra were measured using a Bruker EMX nano X-band (9-GHz) spectrometer using a 0.4-mT modulation amplitude, a 100-kHz modulation frequency, and a 0.3-mW microwave power. Sealed capillaries containing the solid samples were placed in a standard 4-mm quartz sample tube. The temperature of the sample was regulated at 100 K and maintained throughout the measurement by a nitrogen evaporator thermostat. One-dimensional field/sweep experiments were acquired using the standard transition metal settings included in the Bruker instrument library: The instrument field was centered at 3200 G, the sweep width was set to 5600 G, and the time constant was set to 4.29 ms. The EPR spectra were measured with an attenuation of 25 dB (∼20 mW) to avoid microwave saturation of the resonance absorption curve. The number of scans was typically set to 64 to improve the signal-to-noise ratio. The Xenon software (Bruker) was used for baseline correction.

### Computational details for DFT calculations

All the calculations were performed using the ORCA quantum chemistry package 5.0.2 (*78*). The geometry optimizations of (HOSiO_3_)Ru(OOCH)(NH_3_)*_n_* (*n* = 1 or 2) were performed at hybrid functional M062X level of theory using the def2-TZVPP basis set (*79*). The vibrational frequencies and IR intensities were subsequently computed at the same levels of theory. All the optimized geometries were verified as minima by the absence of imaginary frequencies.

### Solid-state NMR spectroscopy

The ^1^H solid-state NMR spectrum was recorded on an 800-MHz Bruker spectrometer (18.8 T) equipped with an Avance Neo console and a 1.3-mm HCN three-channel cross-polarization magic angle spinning (CPMAS) probe head. The sample was packed into a 1.3-mm zirconia rotor under ambient conditions and spun at 50-kHz spinning speed using dry nitrogen gas. ^1^H chemical shifts were referenced relative to tetramethylsilane using the secondary reference adamantane at 1.82 ppm (*80*). Pulses (90°) were set to 2.6 μs. Sixteen transients were accumulated, and 5*T_1_ was used as recycling delay.

## References

[1] J.-P. Lange, Towards circular carbo-chemicals – The metamorphosis of petrochemicals. Energy Environ. Sci. 14, 4358–4376 (2021).

[2] J. Bierhals, “Carbon monoxide” in *Ullmann’s Encyclopedia of Industrial Chemistry* (Wiley-VCH Verlag GmbH & Co. KGaA, 2001), vol. 17245, pp. 1–26.

[3] C. Le Berre, P. Serp, P. Kalck, G. P. Torrence, “Acetic acid” in *Ullmann’s Encyclopedia of Industrial Chemistry* (Wiley-VCH Verlag GmbH & Co. KGaA, 2014), vol. 74, pp. 1–34.

[4] J. Ott, V. Gronemann, F. Pontzen, E. Fiedler, G. Grossmann, D. B. Kersebohm, G. Weiss, C. Witte, “Methanol” in *Ullmann’s Encyclopedia of Industrial Chemistry* (Wiley-VCH Verlag GmbH & Co. KGaA, 2012).

[5] M. Pérez-Fortes, J. C. Schöneberger, A. Boulamanti, G. Harrison, E. Tzimas, Formic acid synthesis using CO_2_ as raw material: Techno-economic and environmental evaluation and market potential. Int. J. Hydrogen Energy 41, 16444–16462 (2016).

[6] M. Pérez-Fortes, J. C. Schöneberger, A. Boulamanti, E. Tzimas, Methanol synthesis using captured CO_2_ as raw material: Techno-economic and environmental assessment. Appl. Energy 161, 718–732 (2016).

[7] S. Pratschner, F. Radosits, A. Ajanovic, F. Winter, Techno-economic assessment of a power-to-green methanol plant. J. CO2 Util. 75, 102563 (2023).

[8] R. P. Ye, J. Ding, W. Gong, M. D. Argyle, Q. Zhong, Y. Wang, C. K. Russell, Z. Xu, A. G. Russell, Q. Li, M. Fan, Y. G. Yao, CO_2_ hydrogenation to high-value products via heterogeneous catalysis. Nat. Commun. 10, 5698 (2019). 31836709 10.1038/s41467-019-13638-9 PMC6910949

[9] K. Lee, H. Yan, Q. Sun, Z. Zhang, N. Yan, Mechanism-guided catalyst design for CO_2_ hydrogenation to formate and methanol. Acc. Mater. Res. 4, 746–757 (2023).

[10] S. Mirzakhani, B. H. Yin, M. Masteri-Farahani, A. C. K. Yip, Heterogeneous catalytic systems for carbon dioxide hydrogenation to value-added chemicals. ChemPlusChem 88, e202300157 (2023). 37263976 10.1002/cplu.202300157

[11] J. Hietala, A. Vuori, P. Johnsson, I. Pollari, W. Reutemann, H. Kieczka, “Formic acid” in *Ullmann’s Encyclopedia of Industrial Chemistry* (Wiley-VCH Verlag GmbH & Co. KGaA, 2016), vol. 1, pp. 1–22.

[12] J. S. Lee, J. C. Kim, Y. G. Kim, Methyl formate as a new building block in C1 chemistry. Appl. Catal. 57, 1–30 (1990).

[13] G. Jenner, Homogeneous catalytic reactions involving methyl formate. Appl. Catal. Gen. 121, 25–44 (1995).

[14] R. Sang, Z. Wei, Y. Hu, E. Alberico, D. Wei, X. Tian, P. Ryabchuk, A. Spannenberg, R. Razzaq, R. Jackstell, J. Massa, P. Sponholz, H. Jiao, H. Junge, M. Beller, Methyl formate as a hydrogen energy carrier. Nat. Catal. 6, 543–550 (2023).

[15] H. Konishi, T. Ueda, T. Muto, K. Manabe, Remarkable improvement achieved by imidazole derivatives in ruthenium-catalyzed hydroesterification of alkenes using formates. Org. Lett. 14, 4722–4725 (2012). 22934690 10.1021/ol301850y

[16] R. L. Pruett, R. T. Kacmarcik, Reactions of formic acid. 1. The iridium-catalyzed synthesis of acetic acid from methyl formate. Organometallics 1, 1693–1699 (1982).

[17] M. Lütgendorf, E. O. Elvevoll, M. Röper, Activity of ruthenium/iodine catalysts for the carbonylation of esters to give carboxylic acids. J. Organomet. Chem. 289, 97–106 (1985).

[18] M. Cheong, S. H. Lee, J. C. Kim, J. S. Lee, Y. G. Kim, Nickel-catalysed isomerization of methyl formate to acetic acid. J. Chem. Soc. Chem. Commun. 661–662, 661 (1990).

[19] G. Jenner, Catalyst and solvent effects on the synthesis of acetic acid from methyl formate. Tetrahedron Lett. 31, 3887–3890 (1990).

[20] P. Jürling-Will, T. Botz, G. Franciò, W. Leitner, A “power-to-X” route to acetic acid via palladium-catalyzed isomerization of methyl formate. ChemSusChem 15, e202201006 (2022). 35691934 10.1002/cssc.202201006 PMC9546377

[21] T. Ohnishi, T. Suzuki, T. Yamakawa, S. Shinoda, Isomerization of methyl formate to acetic acid catalysed by the Ru(II)-Sn(II) heteronuclear cluster complex [Ru(SnCl_3_)_5_(PPh_3_)]^3−^. J. Mol. Catal. 84, 51–58 (1993).

[22] J. Yang, P. Wang, H. Neumann, R. Jackstell, M. Beller, Industrially applied and relevant transformations of 1,3-butadiene using homogeneous catalysts. Ind. Chem. Mater. 1, 155–174 (2023).

[23] K. M. K. Yu, C. M. Y. Yeung, S. C. Tsang, Carbon dioxide fixation into chemicals (methyl formate) at high yields by surface coupling over a Pd/Cu/ZnO nanocatalyst. J. Am. Chem. Soc. 129, 6360–6361 (2007). 17465547 10.1021/ja0706302

[24] K. M. Kerry Yu, S. C. Tsang, A study of methyl formate production from carbon dioxide hydrogenation in methanol over a copper zinc oxide catalyst. Catal. Lett. 141, 259–265 (2011).

[25] C. Wu, Z. Zhang, Q. Zhu, H. Han, Y. Yang, B. Han, Highly efficient hydrogenation of carbon dioxide to methyl formate over supported gold catalysts. Green Chem. 17, 1467–1472 (2015).

[26] J. J. Corral-Pérez, A. Bansode, C. S. Praveen, A. Kokalj, H. Reymond, A. Comas-Vives, J. VandeVondele, C. Copéret, P. R. von Rohr, A. Urakawa, Decisive role of perimeter sites in silica-supported Ag nanoparticles in selective hydrogenation of CO_2_ to methyl formate in the presence of methanol. J. Am. Chem. Soc. 140, 13884–13891 (2018). 30269494 10.1021/jacs.8b08505

[27] H. Reymond, J. J. Corral-Pérez, A. Urakawa, P. R. von Rohr, Towards a continuous formic acid synthesis: A two-step carbon dioxide hydrogenation in flow. React. Chem. Eng. 3, 912–919 (2018).

[28] J. J. Corral-Pérez, C. Copéret, A. Urakawa, Lewis acidic supports promote the selective hydrogenation of carbon dioxide to methyl formate in the presence of methanol over Ag catalysts. J. Catal. 380, 153–160 (2019).

[29] R. Sun, A. Kann, H. Hartmann, A. Besmehn, P. J. C. Hausoul, R. Palkovits, Direct synthesis of methyl formate from CO_2_ with phosphine-based polymer-bound Ru catalysts. ChemSusChem 12, 3278–3285 (2019). 31034754 10.1002/cssc.201900808

[30] H. Park, K. Park, K.-D. Jung, S. Yoon, CO_2_ hydrogenation into formate and methyl formate using Ru molecular catalysts supported on NNN pincer porous organic polymers. Inorg. Chem. Front. 8, 1727–1735 (2021).

[31] O. Kröcher, R. A. Köppel, M. Fröba, A. Baiker, Silica hybrid gel catalysts containing group(VIII) transition metal complexes: Preparation, structural, and catalytic properties in the synthesis of*N*,*N*-dimethylformamide and methyl formate from supercritical carbon dioxide. J. Catal. 178, 284–298 (1998).

[32] P. G. Jessop, Y. Hsiao, T. Ikariya, R. Noyori, Homogeneous catalysis in supercritical fluids: Hydrogenation of supercritical carbon dioxide to formic acid, alkyl formates, and formamides. J. Am. Chem. Soc. 118, 344–355 (1996).

[33] C. A. Huff, M. S. Sanford, Cascade catalysis for the homogeneous hydrogenation of CO_2_ to methanol. J. Am. Chem. Soc. 133, 18122–18125 (2011). 22029268 10.1021/ja208760j

[34] K. Thenert, K. Beydoun, J. Wiesenthal, W. Leitner, J. Klankermayer, Ruthenium-catalyzed synthesis of dialkoxymethane ethers utilizing carbon dioxide and molecular hydrogen. Angew. Chem. Int. Ed. Engl. 55, 12266–12269 (2016). 27581330 10.1002/anie.201606427

[35] M. Siebert, M. Seibicke, A. F. Siegle, S. Kräh, O. Trapp, Selective ruthenium-catalyzed transformation of carbon dioxide: An alternative approach toward formaldehyde. J. Am. Chem. Soc. 141, 334–341 (2019). 30525577 10.1021/jacs.8b10233

[36] N. Westhues, M. Belleflamme, J. Klankermayer, Base-free hydrogenation of carbon dioxide to methyl formate with a molecular ruthenium-phosphine catalyst. ChemCatChem 11, 5269–5274 (2019).

[37] M. Scott, C. G. Westhues, T. Kaiser, J. C. Baums, A. Jupke, G. Franciò, W. Leitner, Methyl formate from CO_2_: An integrated process combining catalytic hydrogenation and reactive distillation. Green Chem. 21, 6307–6317 (2019).

[38] X. Cui, W. Li, P. Ryabchuk, K. Junge, M. Beller, Bridging homogeneous and heterogeneous catalysis by heterogeneous single-metal-site catalysts. Nat. Catal. 1, 385–397 (2018).

[39] B. Fan, M. Jiang, G. Wang, Y. Zhao, B. Mei, J. Han, L. Ma, C. Li, G. Hou, T. Wu, L. Yan, Y. Ding, Elucidation of hemilabile-coordination-induced tunable regioselectivity in single-site Rh-catalyzed heterogeneous hydroformylation. Nat. Commun. 15, 6967 (2024). 39138177 10.1038/s41467-024-51281-1 PMC11322285

[40] A. D. Allen, F. Bottomley, R. O. Harris, V. P. Reinsalu, C. V. Senoff, Ruthenium complexes containing molecular nitrogen. J. Am. Chem. Soc. 89, 5595–5599 (1967).

[41] C. P. Madhusudhan, M. D. Patil, M. L. Good, Chemical reactivity of ruthenium complexes supported on Y-type zeolites. Inorg. Chem. 18, 2384–2389 (1979).

[42] P. Castillo-Villalón, J. Ramírez, M.-J. Peltre, C. Louis, P. Massiani, An UV-Visible study of the stability of the ruthenium hexaammine cation in BEA zeolites—Comparison with NaY. Phys. Chem. Chem. Phys. 6, 3739–3746 (2004).

[43] J. R. Pearce, B. L. Gustafson, J. H. Lunsford, Characterization of nitrosyl intermediates formed during the decomposition of hexaammineruthenium(3+) complexes in zeolites. Inorg. Chem. 20, 2957–2960 (1981).

[44] M. Newville, J. J. Kas, J. J. Rehr, Improvements in modeling EXAFS with many-pole self-energy and FEFF 8.5. J. Phys. Conf. Ser. 190, 012023 (2009).

[45] M. M. J. Treacy, J. B. Higgins, *Collection of Simulated XRD Powder Patterns for Zeolites* (Elsevier, ed. 5, 2007); https://linkinghub.elsevier.com/retrieve/pii/B9780444530677X54707.

[46] D. Freude, M. Hunger, H. Pfeifer, Investigation of acidic properties of zeolites by MAS NMR. Z. Für Phys. Chem. 152, 171–182 (1987).

[47] M. Hunger, Br⊘nsted acid sites in zeolites characterized by multinuclear solid-state NMR spectroscopy. Catal. Rev. 39, 345–393 (1997).

[48] T. Tatsumi, W. Maekawa, H. Tominaga, A study of the thermal decomposition of ruthenium ammine zeolites. Bull. Chem. Soc. Jpn. 57, 579–580 (1984).

[49] J. R. Pearce, W. J. Mortier, J. B. Uytterhoeven, Oxidation–reduction behaviour of highly dispersed ruthenium in RuNaY zeolite. J. Chem. Soc. Faraday 75, 1395–1402 (1979).

[50] M. Goldwasser, J. F. Dutel, C. Naccache, E.s.r. and i.r. studies of ruthenium (III) hexaamine-exchanged zeolites. Zeolites 9, 54–58 (1989).

[51] M. A. A. Aziz, A. A. Jalil, S. Triwahyono, A. Ahmad, CO_2_ methanation over heterogeneous catalysts: Recent progress and future prospects. Green Chem. 17, 2647–2663 (2015).

[52] F. Wang, S. He, H. Chen, B. Wang, L. Zheng, M. Wei, D. G. Evans, X. Duan, Active site dependent reaction mechanism over Ru/CeO_2_ catalyst toward CO_2_ methanation. J. Am. Chem. Soc. 138, 6298–6305 (2016). 27135417 10.1021/jacs.6b02762

[53] T. Sakpal, L. Lefferts, Structure-dependent activity of CeO_2_ supported Ru catalysts for CO_2_ methanation. J. Catal. 367, 171–180 (2018).

[54] T. Siudyga, M. Kapkowski, P. Bartczak, M. Zubko, J. Szade, K. Balin, S. Antoniotti, J. Polanski, Ultra-low temperature carbon (di)oxide hydrogenation catalyzed by hybrid ruthenium-nickel nanocatalysts: Towards sustainable methane production. Green Chem. 22, 5143–5150 (2020).

[55] S. F. Yin, B. Q. Xu, X. P. Zhou, C. T. Au, A mini-review on ammonia decomposition catalysts for on-site generation of hydrogen for fuel cell applications. Appl. Catal. Gen. 277, 1–9 (2004).

[56] K. N. Heck, M. O. Nutt, P. Alvarez, M. S. Wong, Deactivation resistance of Pd/Au nanoparticle catalysts for water-phase hydrodechlorination. J. Catal. 267, 97–104 (2009).

[57] J. Li, M. Kitano, T.-N. Ye, M. Sasase, T. Yokoyama, H. Hosono, Chlorine-tolerant ruthenium catalyst derived using the unique anion-exchange properties of 12CaO·7Al_2_O_3_ for ammonia synthesis. ChemCatChem 9, 3078–3083 (2017).

[58] A. Boddien, D. Mellmann, F. Gärtner, R. Jackstell, H. Junge, P. J. Dyson, G. Laurenczy, R. Ludwig, M. Beller, Efficient dehydrogenation of formic acid using an iron catalyst. Science 333, 1733–1736 (2011). 21940890 10.1126/science.1206613

[59] M. J. Drummond, T. J. Miller, C. L. Ford, A. R. Fout, Catalytic perchlorate reduction using iron: Mechanistic insights and improved catalyst turnover. ACS Catal. 10, 3175–3182 (2020).

[60] M. A. Ehweiner, F. Wiedemaier, B. Lajin, J. A. Schachner, F. Belaj, W. Goessler, N. C. Mösch-Zanetti, Nature-inspired homogeneous catalytic perchlorate reduction using molybdenum complexes. ACS Catal. 11, 11754–11761 (2021).

[61] E. J. Beckman, Supercritical and near-critical CO_2_ in green chemical synthesis and processing. J. Supercrit. Fluids 28, 121–191 (2004).

[62] M. Zhang, M. Dou, M. Wang, Y. Yu, Study on the solubility parameter of supercritical carbon dioxide system by molecular dynamics simulation. J. Mol. Liq. 248, 322–329 (2017).

[63] G. Garg, M. Gómez, A. M. Masdeu-Bultó, Y. Medina González, Compressed carbon dioxide as a medium in catalytic hydrogenations: Engineering and chemistry. J. CO2 Util. 77, 102605 (2023).

[64] A. C. Deacy, E. Moreby, A. Phanopoulos, C. K. Williams, Co(III)/alkali-metal(I) heterodinuclear catalysts for the ring-opening copolymerization of CO_2_ and propylene oxide. J. Am. Chem. Soc. 142, 19150–19160 (2020). 33108736 10.1021/jacs.0c07980 PMC7662907

[65] R. Linnell, Notes- Dissociation constants of 2-substituted pyridines. J. Org. Chem. 25, 290–290 (1960).

[66] B. Lenarcik, P. Ojczenasz, The influence of the size and position of the alkyl groups in alkylimidazole molecules on their acid-base properties. J. Heterocyclic Chem. 39, 287–290 (2002).

[67] S. Tshepelevitsh, A. Kütt, M. Lõkov, I. Kaljurand, J. Saame, A. Heering, P. G. Plieger, R. Vianello, I. Leito, On the basicity of organic bases in different media. Eur. J. Org. Chem. 2019, 6735–6748 (2019).

[68] O. Ben Mya, M. Bita, I. Louafi, A. Djouadi, Esterification process catalyzed by ZSM-5 zeolite synthesized via modified hydrothermal method. MethodsX 5, 277–282 (2018). 30038897 10.1016/j.mex.2018.03.004 PMC6053634

[69] N. Fattahi, K. Triantafyllidis, R. Luque, A. Ramazani, Zeolite-based catalysts: A valuable approach toward ester bond formation. Catalysts 9, 758 (2019).

[70] G. J. Gomes, M. F. Zalazar, J. C. Padilha, M. B. Costa, C. L. Bazzi, P. A. Arroyo, Unveiling the mechanisms of carboxylic acid esterification on acid zeolites for biomass-to-energy: A review of the catalytic process through experimental and computational studies. Chemosphere 349, 140879 (2024). 38061565 10.1016/j.chemosphere.2023.140879

[71] G. J. Gomes, D. M. Dal Pozzo, M. F. Zalazar, M. B. Costa, P. A. Arroyo, P. R. S. Bittencourt, Oleic acid esterification catalyzed by zeolite Y-model of the biomass conversion. Top. Catal. 62, 874–883 (2019).

[72] P. Salvador, W. Kladnig, Surface reactivity of zeolites type H-Y and Na-Y with methanol. J. Chem. Soc. Faraday 73, 1153 (1977).

[73] D. H. Gibson, Y. Ding, R. L. Miller, B. A. Sleadd, M. S. Mashuta, J. F. Richardson, Synthesis and characterization of ruthenium, rhenium and titanium formate, acetate and trifluoroacetate complexes. Correlation of IR spectral properties and bonding types. Polyhedron 18, 1189–1200 (1999).

[74] X. Huo, D. J. Van Hoomissen, J. Liu, S. Vyas, T. J. Strathmann, Hydrogenation of aqueous nitrate and nitrite with ruthenium catalysts. Appl. Catal. Environ. 211, 188–198 (2017).

[75] S. Moret, P. J. Dyson, G. Laurenczy, Direct synthesis of formic acid from carbon dioxide by hydrogenation in acidic media. Nat. Commun. 5, 1–7 (2014). 10.1038/ncomms5017 PMC4059918 24886955

[76] M. Newville, Larch: An analysis package for XAFS and related spectroscopies. J. Phys. Conf. Ser. 430, 012007 (2013).

[77] A. L. Ankudinov, B. Ravel, J. J. Rehr, S. D. Conradson, Real-space multiple-scattering calculation and interpretation of x-ray-absorption near-edge structure. Phys. Rev. B 58, 7565–7576 (1998).

[78] F. Neese, Software update: The ORCA program system—Version 5.0. WIREs Comput. Mol. Sci. 12, e1606 (2022).

[79] Y. Zhao, D. G. Truhlar, The M06 suite of density functionals for main group thermochemistry, thermochemical kinetics, noncovalent interactions, excited states, and transition elements: Two new functionals and systematic testing of four M06-class functionals and 12 other functionals. Theor. Chem. Acc. 120, 215–241 (2008).

[80] R. K. Harris, E. D. Becker, S. M. C. de Menezes, R. Goodfellow, P. Granger, NMR nomenclature., Nuclear spin properties and conventions for chemical shifts (IUPAC Recommendations 2001). Pure Appl. Chem. 73, 1795–1818 (2001). 10.1006/snmr.2002.0063 12637147

