# Supplementary material for: Single-site ruthenium catalyst supported on zeolite for CO2 hydrogenation to methyl formate
Source: Sci Adv. 2025 Apr 16;11(16):eadu2857. doi: 10.1126/sciadv.adu2857 (PMC12002109; doi:10.1126/sciadv.adu2857)
Supplement: Supplementary file 1 — Figs. S1 to S25 Tables S1 to S6 [file sciadv.adu2857_sm.pdf]

Science Advances
AAAS

Supplementary Materials for

Single-site ruthenium catalyst supported on zeolite for CO_2 hydrogenation to methyl formate

Roland C. Turnell-Ritson *et al.*

Corresponding author: Roland C. Turnell-Ritson, roland.turnell-ritson@epfl.ch; Paul J. Dyson, paul.dyson@epfl.ch

Sci. Adv. **11**, eadu2857 (2025)
DOI: 10.1126/sciadv.adu2857

This PDF file includes:

Figs. S1 to S25
Tables S1 to S6

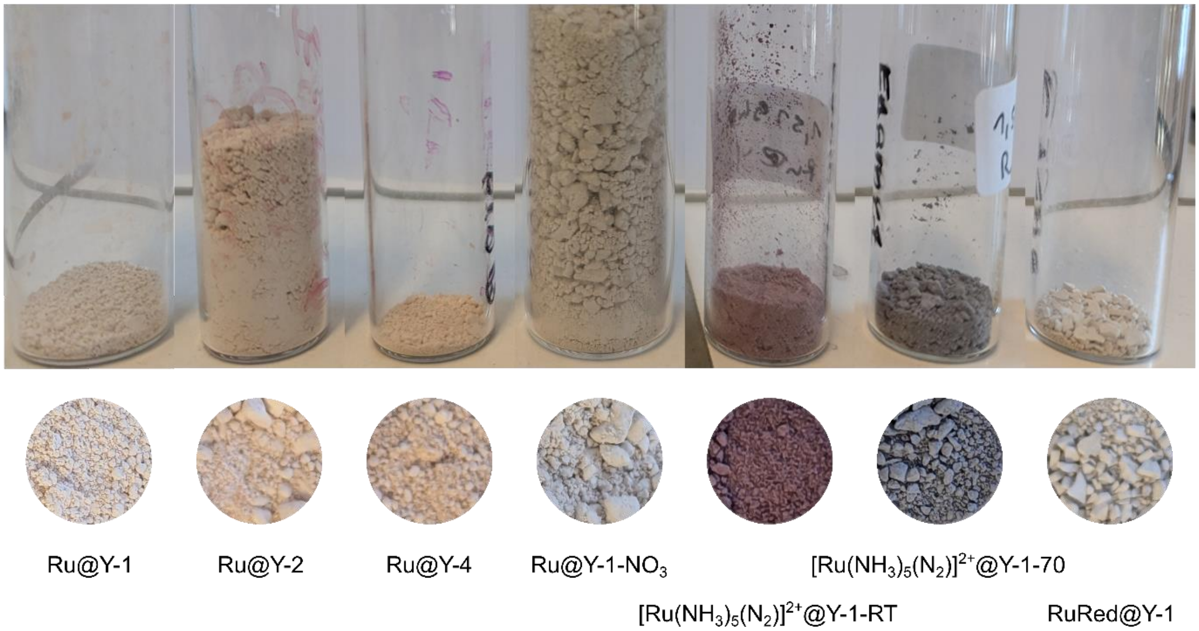

Fig. S1. Catalyst Photographs

Photographs of some of the supported catalysts prepared in this work.

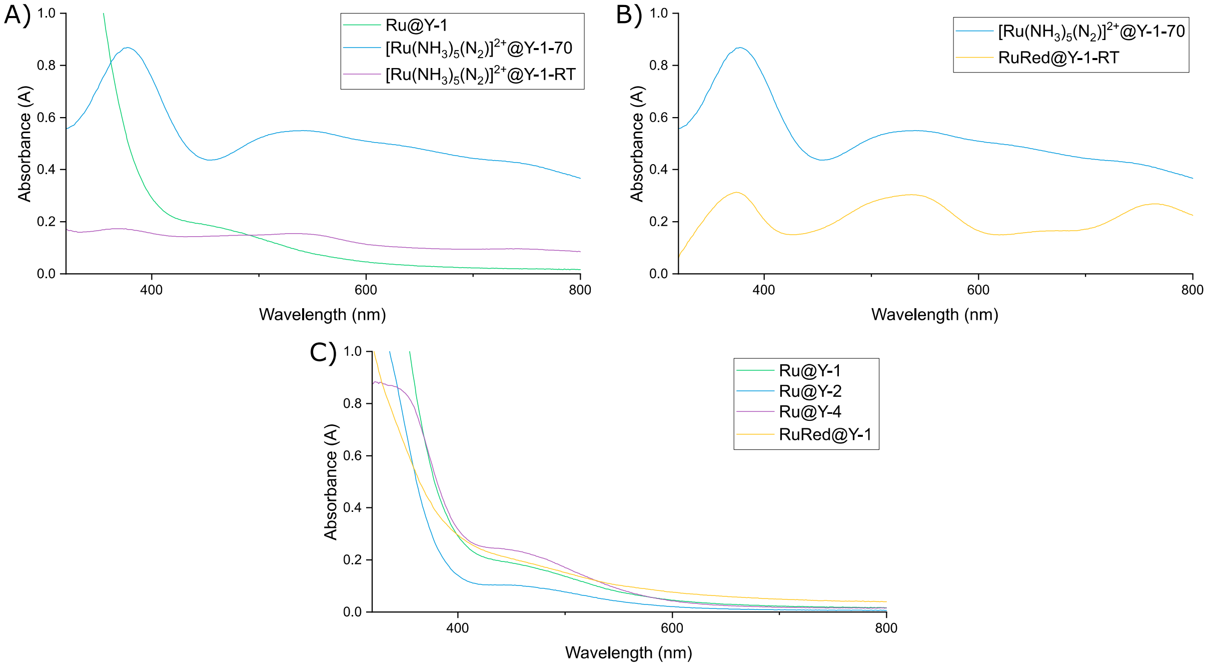

Fig. S2. UV-Vis Spectroscopy

Comparative UV-Vis spectra of: A) $[Ru(NH_3)_5N_2]^{2+}$ freshly exchanged onto NaY ($[Ru(NH_3)_5N_2]^{2+}$@Y-1), the same material after heating to 70°C for 24 h ($[Ru(NH_3)_5N_2]^{2+}$@Y-1-70), and Ru@Y-1; B) $[Ru(NH_3)_5N_2]^{2+}$@Y-1-70, and ruthenium red trimer freshly exchanged onto NaY (RuRed@Y-1-RT); C) three different weight loadings of the catalyst, Ru@Y-1, Ru@Y-2 and Ru@Y-4, and ruthenium red trimer on zeolite Y heated to 180°C overnight (RuRed@Y-1).

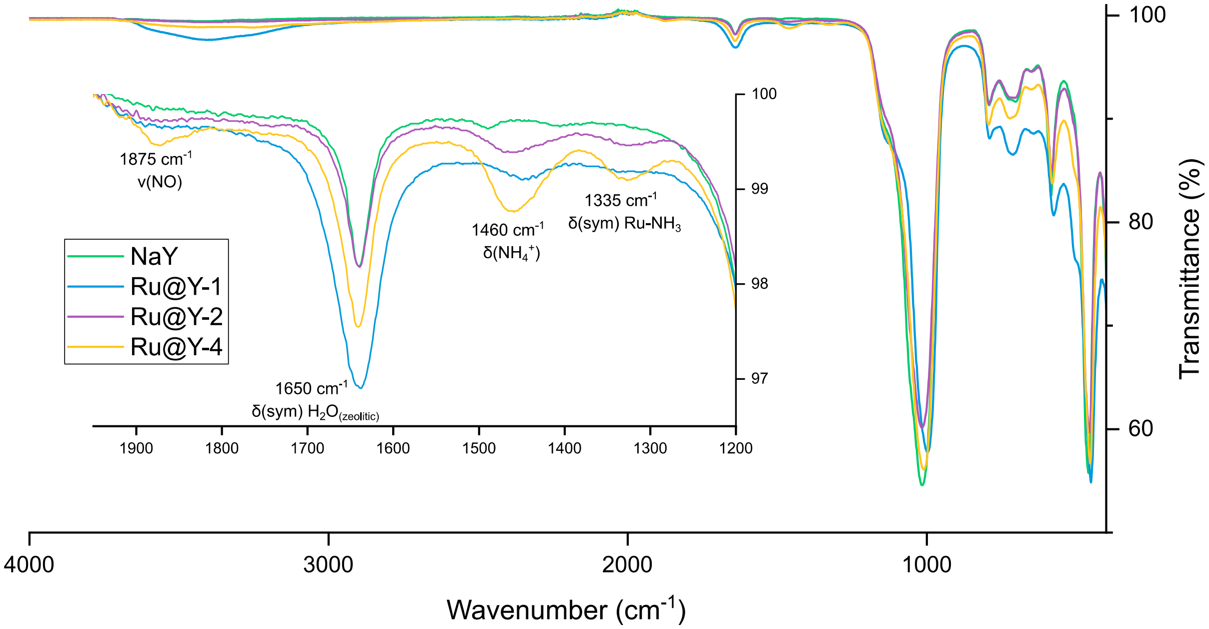

Fig. S3. IR spectroscopy

Transmittance IR spectra of the Ru@Y-x catalysts, with magnified inset of the region 1950-1200 cm^{-1}, and associated peak assignments. (*43*)

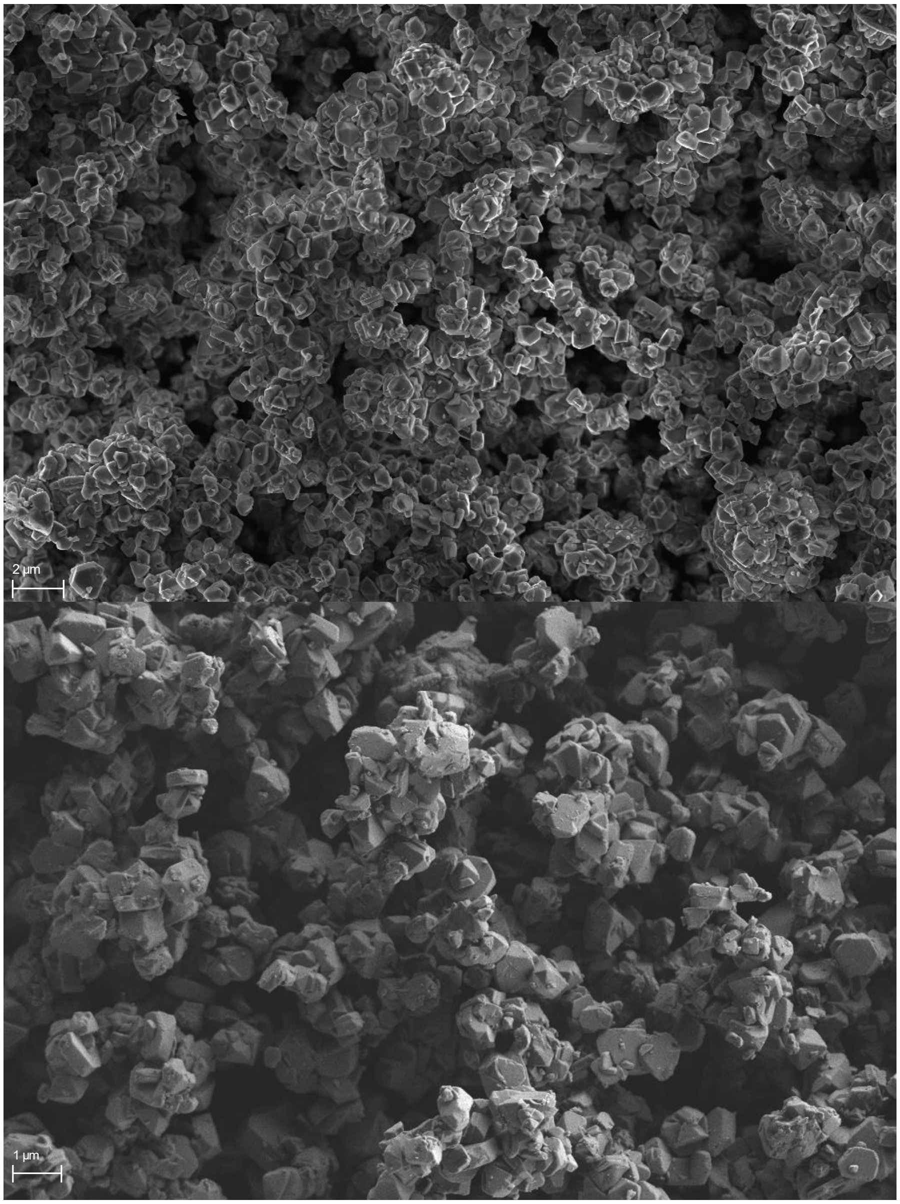

Fig. S4. Scanning Electron Microscopy (SEM)

SEM images of Ru@Y-1, at two different magnifications. Top: working distance (WD) = 2.8 mm, magnification (Mag) = 3.33 kx, InLens secondary electron detector (SE), electron high tension (EHT) = 3.00 keV. Bottom: WD = 5.8 mm, Mag = 6.47 kx, Everhart-Thornley SE, EHT = 1.00 keV.

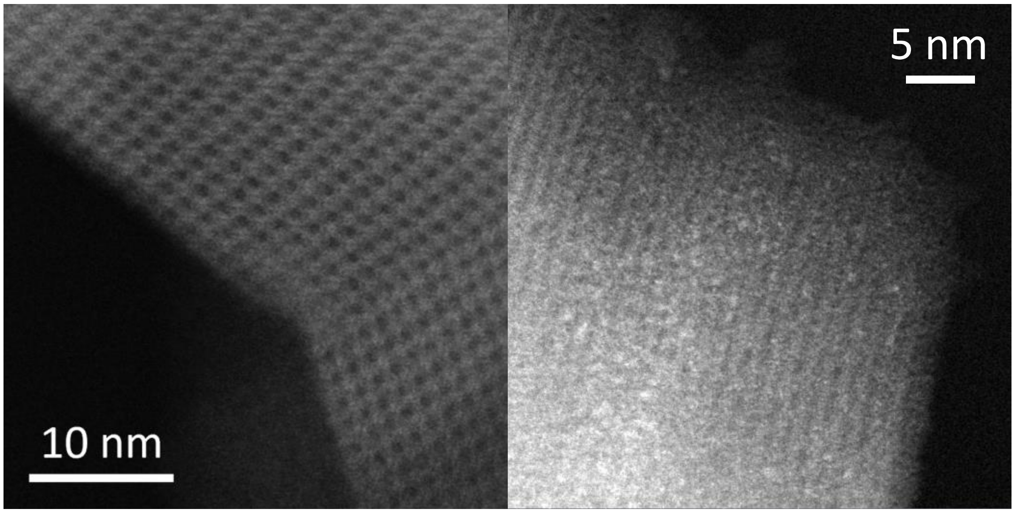

Fig. S5. Annular Dark-Field Scanning Transmission Electron Microscopy (ADF-STEM)
ADF-STEM images of Ru@Y-2, at 1.36 Mx magnification (left), and at 2.72 Mx magnification (right).

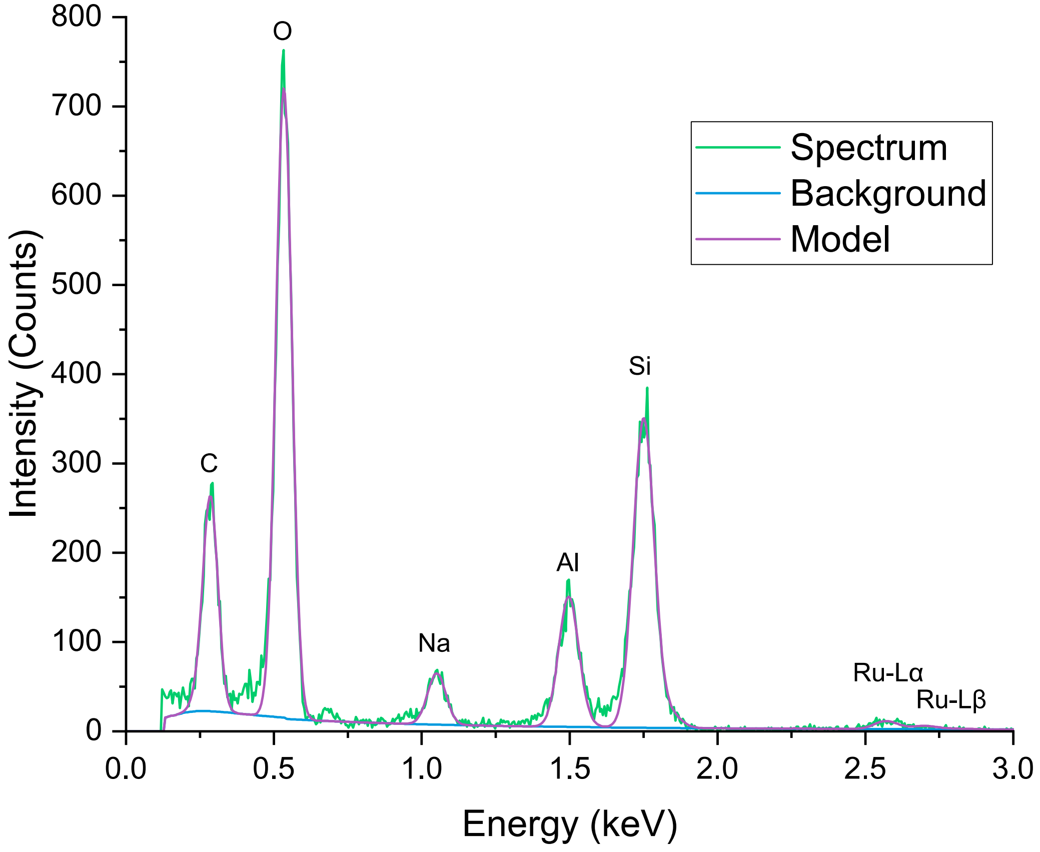

Fig. S6. EDX Spectroscopy

EDX spectrum of the chemical species in a particle of Ru@Y-2.

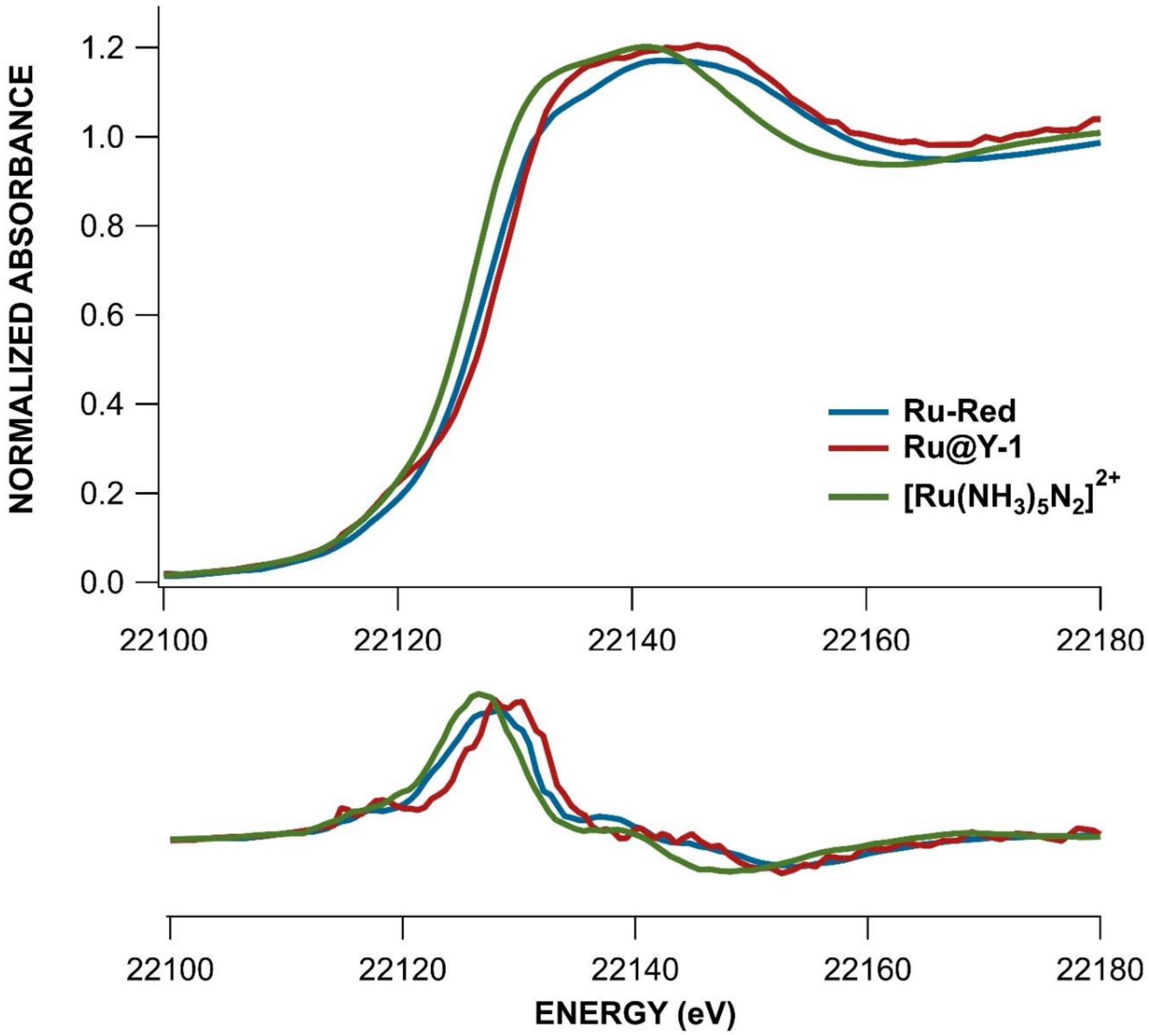

Fig. S7. X-Ray Absorption Spectroscopy (XAS) plots of Ru@Y-1, $[Ru(NH_3)_5N_2]I_2$, and RuRed

XAS plot of Ru@Y-1, $[Ru(NH_3)_5N_2]I_2$, and RuRed (top) and the first derivative for the respective spectra (bottom).

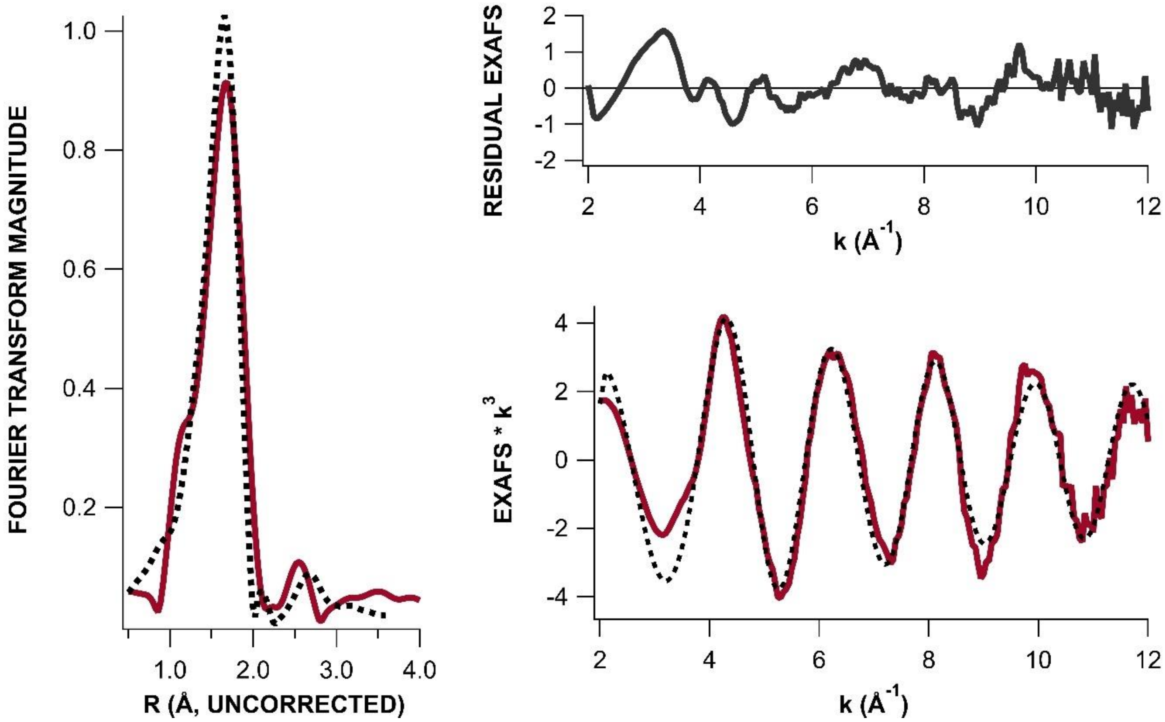

Fig. S8. EXAFS fit of [Ru(NH_3)$_5$$N_2$]$I_2$

EXAFS fit of [Ru(NH_3)$_5$$N_2$]$I_2$. Spectra were fit between 1 < R < 3.

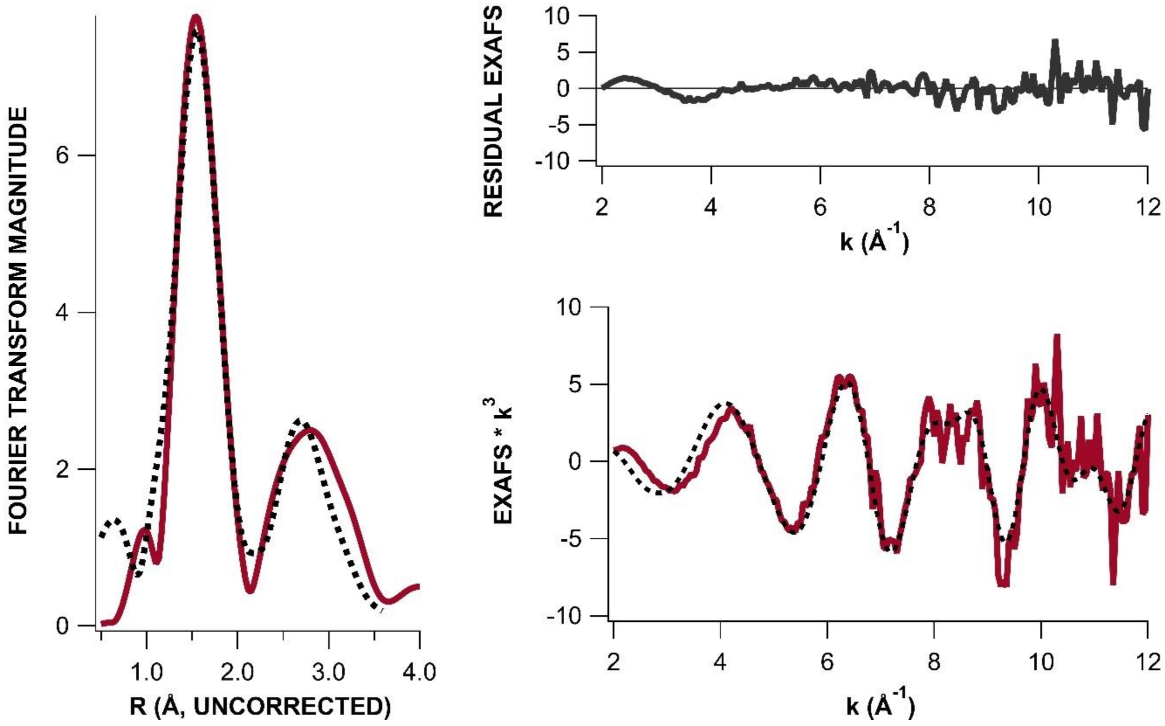

Fig. S9. EXAFS fit of Ru@Y-1

EXAFS fit of Ru@Y-1. The Fourier transform was performed with Kaiser-Bessel window applied between $2 < k < 12$. A model generated from putative binding of the Ru centre to the [6,4] vertex of Zeolite Y was used as the input to the FEFF calculation.(*44*, *77*) While the first shell could be modelled with reasonable fidelity, care should be taken interpreting the second shell (ca. $R > 2.0$) modelling. The Si–O multiple scatter has been modelled with a coordination number of 6 which is almost certainly unphysical – this is to encapsulate the multitude of other multiple scattering paths at a similar distance, instead of modelling each multiple scattering path individually. Similarly, the Ru–O single scatterer found at 3.81 Å and the coordination number of 12 is unphysical – the observed is likely a combination of zeolite O atoms, residual water within pores, and other single- and multiple-scattering paths. Regardless, the second shell appears to be remarkably consistent with the proposed mode of binding.

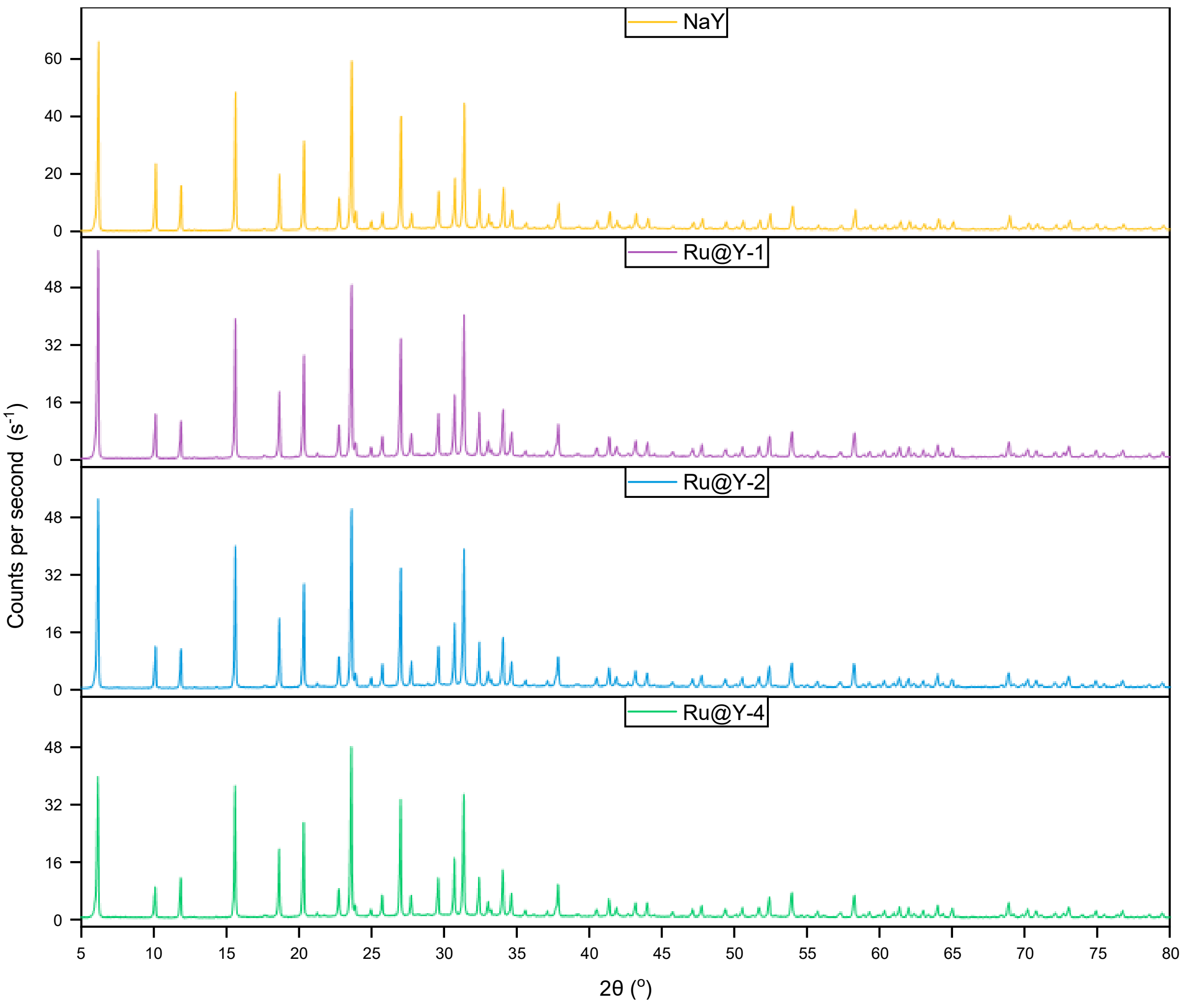

Fig. S10. Powder X-Ray Diffractometry (PXRD)

PXRD patterns of NaY (top) and freshly prepared Ru@Y-x catalysts.

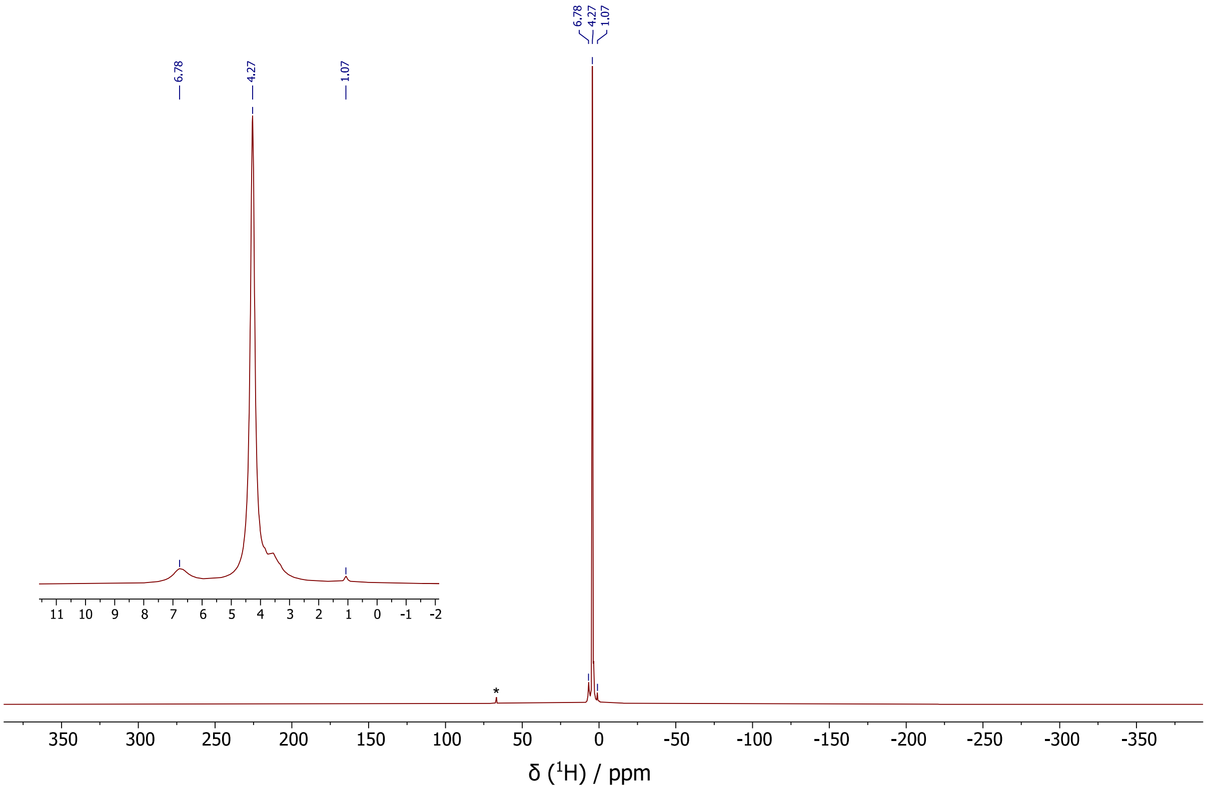

Fig. S11. Solid State Magic Angle Spinning NMR (MAS-NMR) spectroscopy

^{1}H MAS NMR spectrum of Ru@Y-4, with inset of the magnified region between -2 and 11 ppm. * indicates a spinning side band.

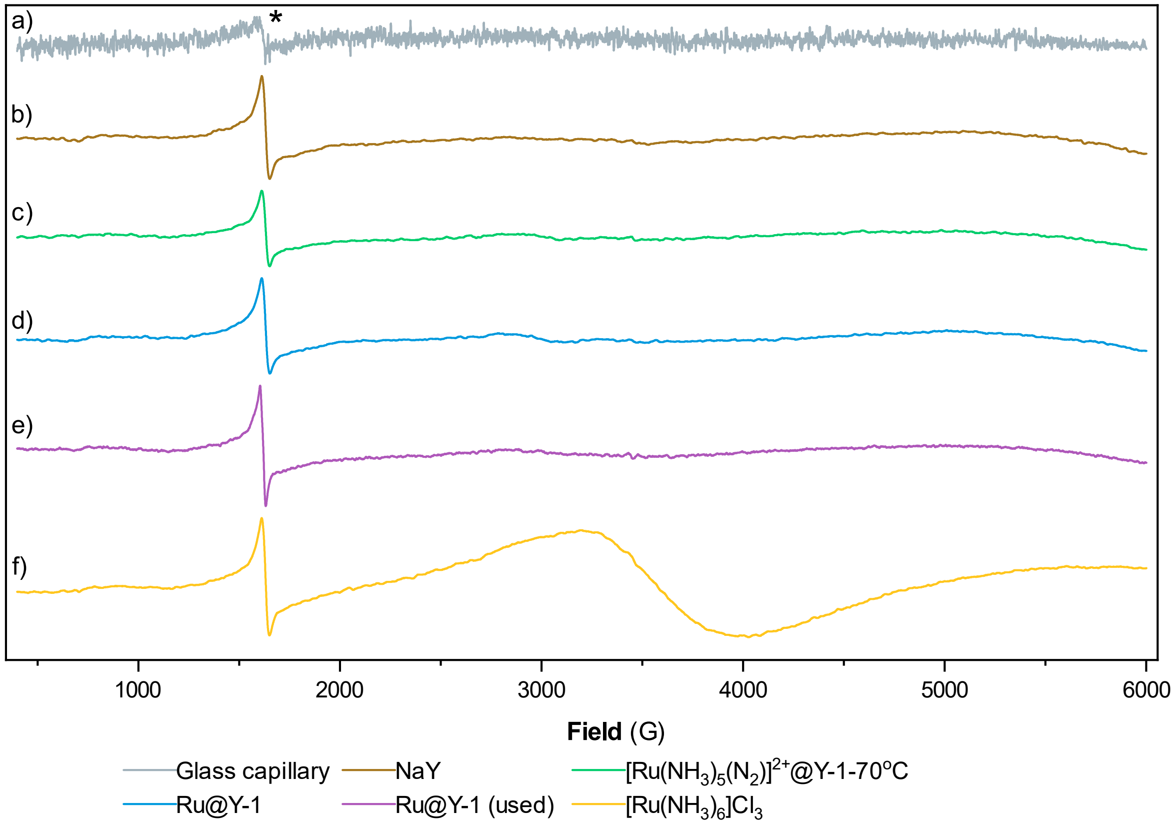

Fig. S12. Electron Paramagnetic Resonance (EPR) spectroscopy

EPR spectra recorded at 100 K, showing the spectrum of a) an empty glass capillary used to contain the samples, which gives a signal in all samples marked by *; b) NaY; c) $[Ru(NH_3)_5N_2]^{2+}$@Y-1-70; d) Ru@Y-1; e) Ru@Y-1 after use; and f) $[Ru(NH_3)_6]Cl_3$, showing the broad peak which would be expected from a paramagnetic Ru(III) species.

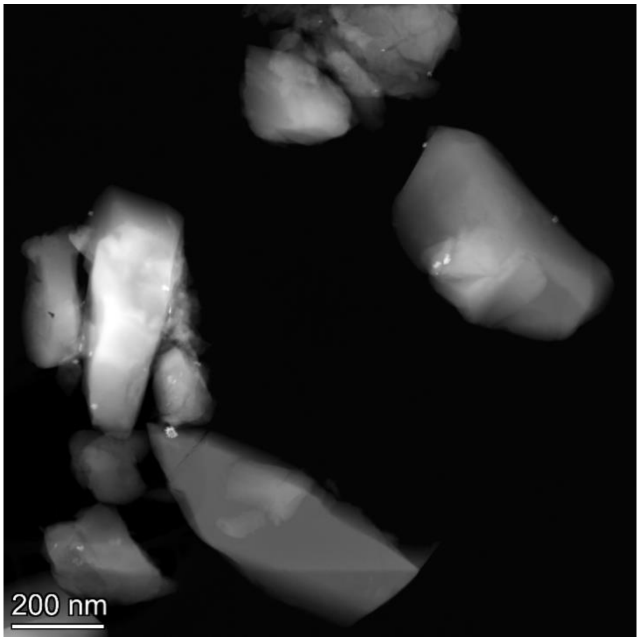

Fig. S13. HAADF-STEM of spent catalyst

HAADF-STEM of spent catalyst, showing the formation of a small number of Ru nanoparticles.

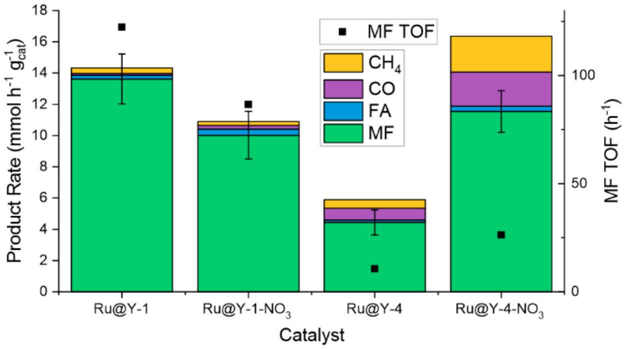

Fig. S14. Optimization: Ru@Y-x-NO_3

Variation in the productivity (left axis) and activity (right axis) from the Ru@Y-x and Ru@Y-x-NO_3 catalyzed methanol hydrocarboxylation as a function of the weight loading (x). Conditions: Catalyst (25 mg), N-^{n}Pr-Im (500 mg), MeOH (40 mL), CO_2 (20 bar), H_2 (80 bar), 160°C, 16 h. Average of at least two runs, with standard error in MF production depicted.

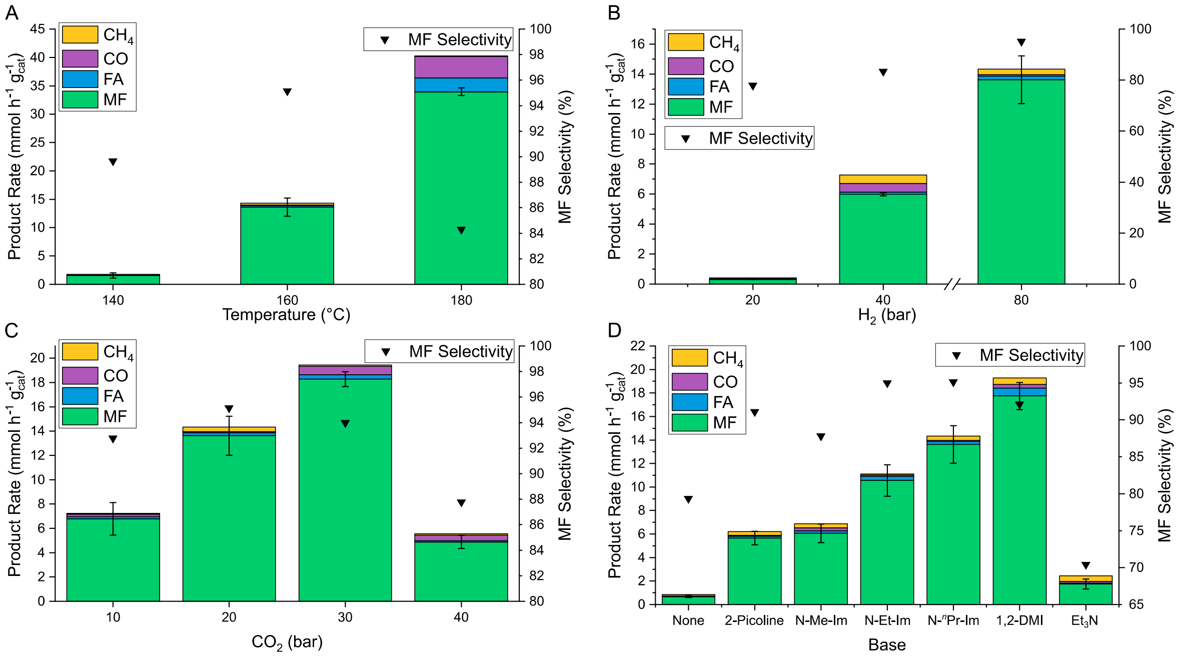

Fig. S15. Optimization: Effect of conditions on the overall productivity

Variation in the productivity (left axis) and selectivity towards MF (right axis) from Ru@Y-1 catalyzed methanol hydrocarboxylation as a function of: **A** Temperature; **B** H_2 pressure; **C** CO_2 pressure; **D** Choice of base (500 mg). Conditions (unless otherwise stated): Ru@Y-1 (25 mg), N-^{n}Pr-Im (500 mg), MeOH (40 mL), CO_2 (20 bar), H_2 (80 bar), 160℃, 16 h. Average of at least two runs, with standard error in MF production depicted.

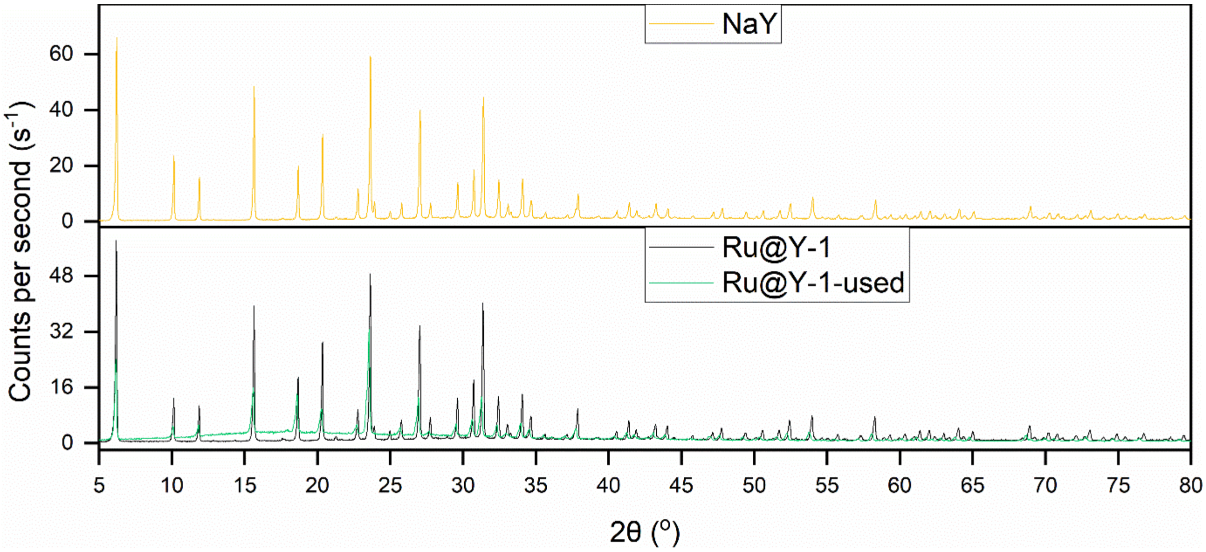

Fig. S16. PXRD of used catalyst

PXRD traces of NaY (top) and freshly prepared Ru@Y-1 overlaid with Ru@Y-1 after use (bottom).

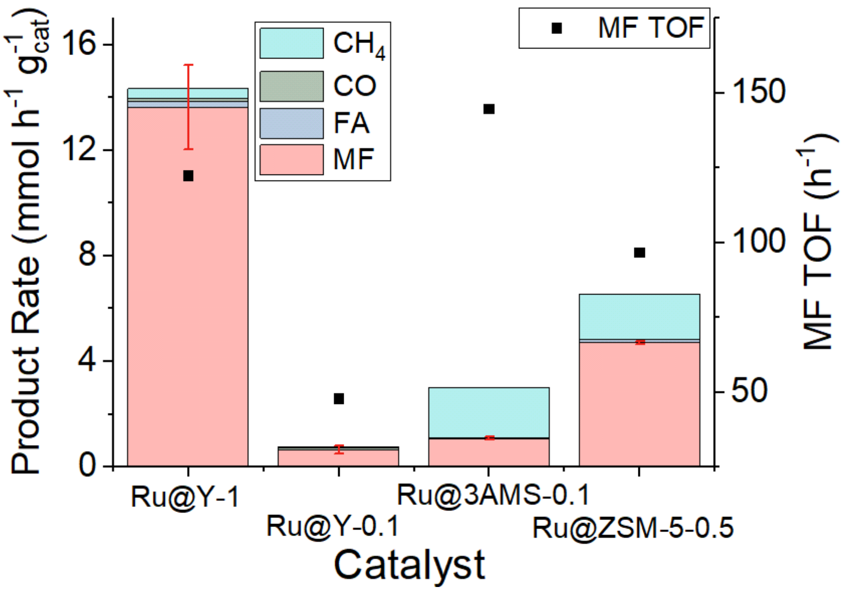

Fig. S17. Support modification

Variation in the productivity (left axis) and activity (right axis) from the Ru@Zeolite-x catalyzed methanol hydrocarboxylation as a function of the weight loading (x) and zeolite support. Conditions: Catalyst (25 mg), N-^{n}Pr-Im (500 mg), MeOH (40 mL), CO_2 (20 bar), H_2 (80 bar), 160°C, 16 h. Average of at least two runs, with standard error in MF production depicted.

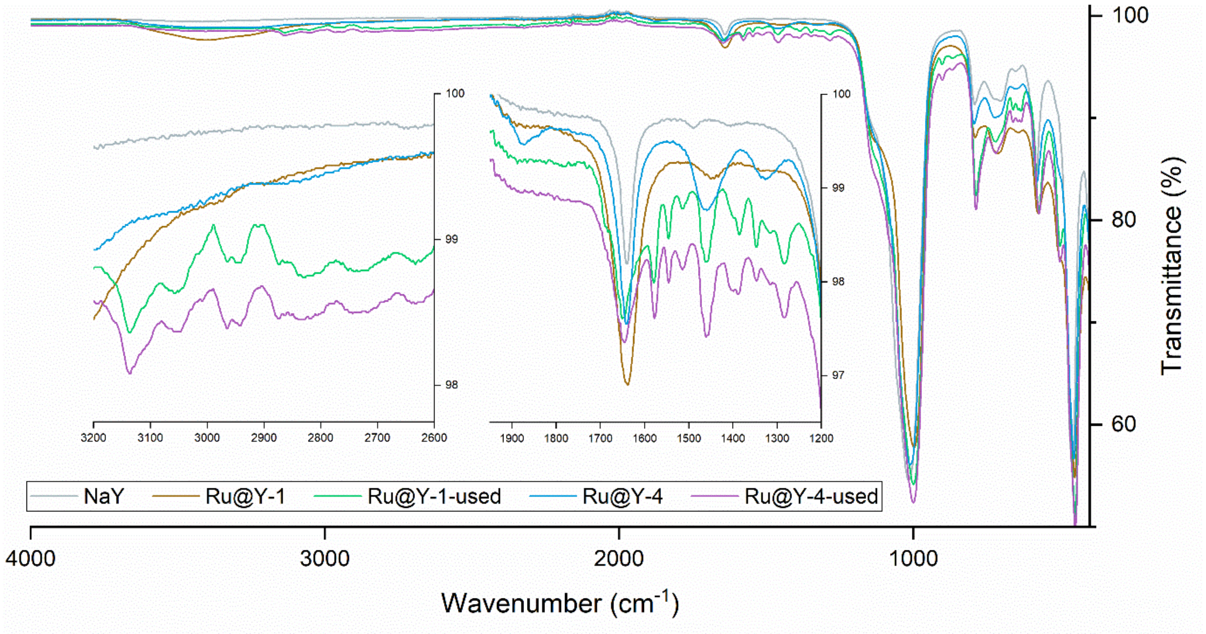

Fig. S18. FTIR spectroscopy of used catalyst (1)

Transmittance IR spectra of NaY and Ru@Y-1 and Ru@Y-4 catalysts, before and after use, with magnified insets of the 3200-2600 cm^{-1} and 1950-1200 cm^{-1} regions.

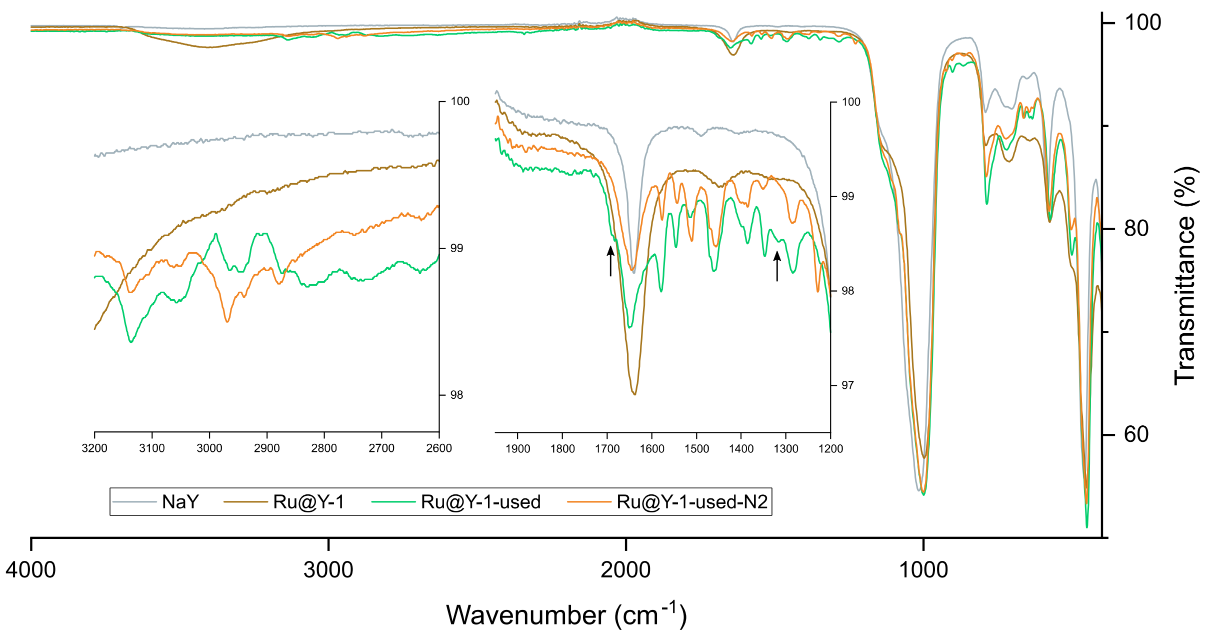

Fig. S19. FTIR spectroscopy of used catalyst (2)

Transmittance IR spectra of NaY and Ru@Y-1, before and after use under CO_2 or N_2, with magnified insets of the 3200-2600 cm^{-1} and 1950-1200 cm^{-1} regions. The arrows indicate differences between the spectra of the used catalysts, indicative of a bidentate OOCH moiety.

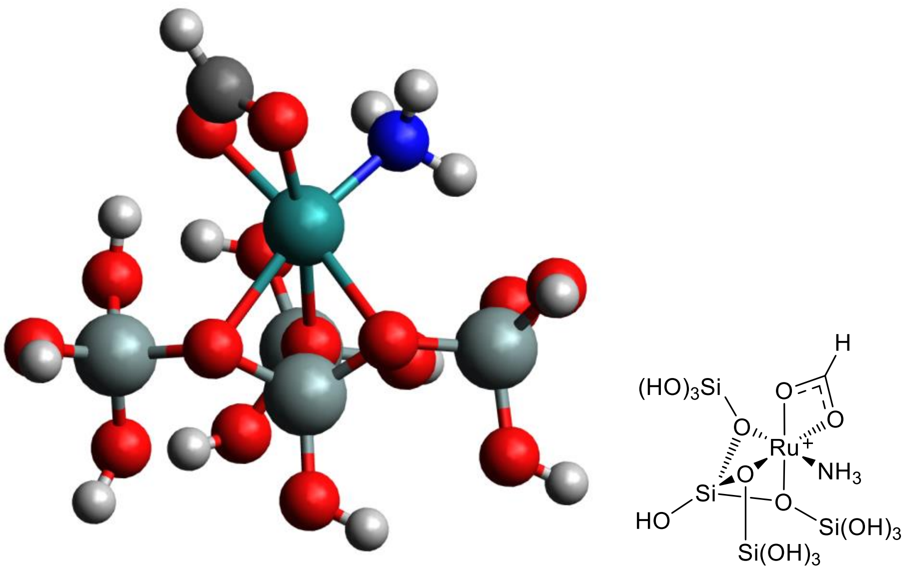

Fig. S20. Optimized Geometry of bidentate Ru-formate: $[(HOSi(OSi(OH)_3)_3)Ru(OOCH)(NH_3)]^+$

Energy = −2483.296348240278 au

Ru	3.938704	12.608816	11.380859
O	4.483685	11.554566	9.444650
O	5.935171	13.399251	10.355455
O	3.486414	13.828224	9.501457
O	4.678722	11.709541	13.140807
N	2.107978	11.733577	11.827149
H	1.597140	12.266240	12.527017
H	2.254454	10.796838	12.196727
H	1.518022	11.655093	10.998772
C	4.318315	12.729006	13.807187
Si	4.913851	13.101949	9.103685
O	5.478738	13.445549	7.657814
H	4.866221	13.733066	6.971190
O	3.743137	13.654902	13.191649
H	4.504843	12.787820	14.881925
Si	2.038489	13.591661	8.703230
O	2.488378	13.933052	7.177013
O	0.894481	14.494833	9.366731
O	1.534906	12.070593	8.912750
Si	4.565997	9.930970	9.141065
O	4.465291	9.158723	10.554527
O	5.867682	9.626305	8.256377
O	3.176606	9.699491	8.324792
Si	7.275466	12.426236	10.658709
O	7.703241	11.828821	9.203971
O	6.822974	11.113298	11.473360
H	0.964402	15.447197	9.443428
H	1.989068	11.311732	8.516690
H	1.853221	13.918277	6.457958
H	2.927490	8.828483	8.009343
H	5.267151	9.048252	11.072574
H	6.361995	11.193883	12.319177
H	8.405780	12.228133	8.687199
O	8.386126	13.298596	11.413784
H	8.425395	14.254764	11.379580
H	6.695427	10.085094	8.443889

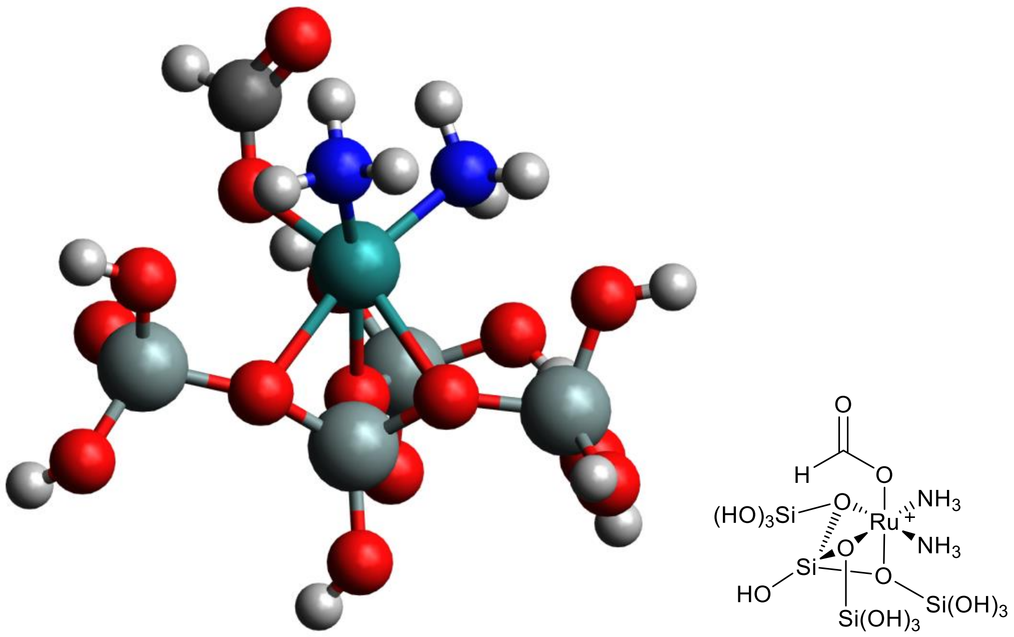

Fig. S21. Optimized Geometry of monodentate Ru-formate: $[(HOSi(OSi(OH)_3)_3)Ru(OOCH)(NH_3)_2]^+$

Energy = −2539.901512656004 au

Ru	4.183201	12.812446	11.021974
O	4.599535	10.908996	9.793988
O	5.915137	13.082527	9.514167
O	3.478561	13.022930	8.788178
O	5.279470	12.154288	12.659586
N	4.104806	14.765368	11.767770
N	2.410354	12.347685	12.033372
H	2.189055	11.354067	11.976921
H	1.620918	12.838544	11.621661
H	2.499493	12.611489	13.013625
H	5.012372	15.201026	11.603248
H	3.960127	14.715713	12.777169
H	3.392655	15.363818	11.362537
C	5.025100	12.532868	13.876123
Si	4.860710	12.118001	8.731271
O	5.186101	11.705561	7.229485
H	5.872409	11.091681	6.962767
O	4.137165	13.292401	14.214119
H	5.698306	12.084759	14.620526
Si	3.887149	9.549821	10.419377
O	4.359169	8.356001	9.447875
O	2.282870	9.803747	10.429018
O	4.358573	9.416471	11.950675
Si	1.946881	12.800208	8.225336
Si	7.383976	13.329063	10.239435
O	8.528168	13.429036	9.112689
O	7.703978	12.100584	11.227442
O	7.099476	14.752380	10.982707
O	1.682809	13.530536	6.818979
O	1.718912	11.206411	7.973121
H	4.874141	10.125031	12.354440
H	1.865564	14.454288	6.645738
H	1.609595	10.900573	7.068777
O	1.044125	13.332379	9.468233
H	0.096505	13.422163	9.342175
H	1.842431	9.940294	9.581645
H	4.324495	7.438010	9.719708
H	9.298539	12.860584	9.157501
H	7.845974	15.274110	11.284071
H	7.071266	11.939168	11.943414

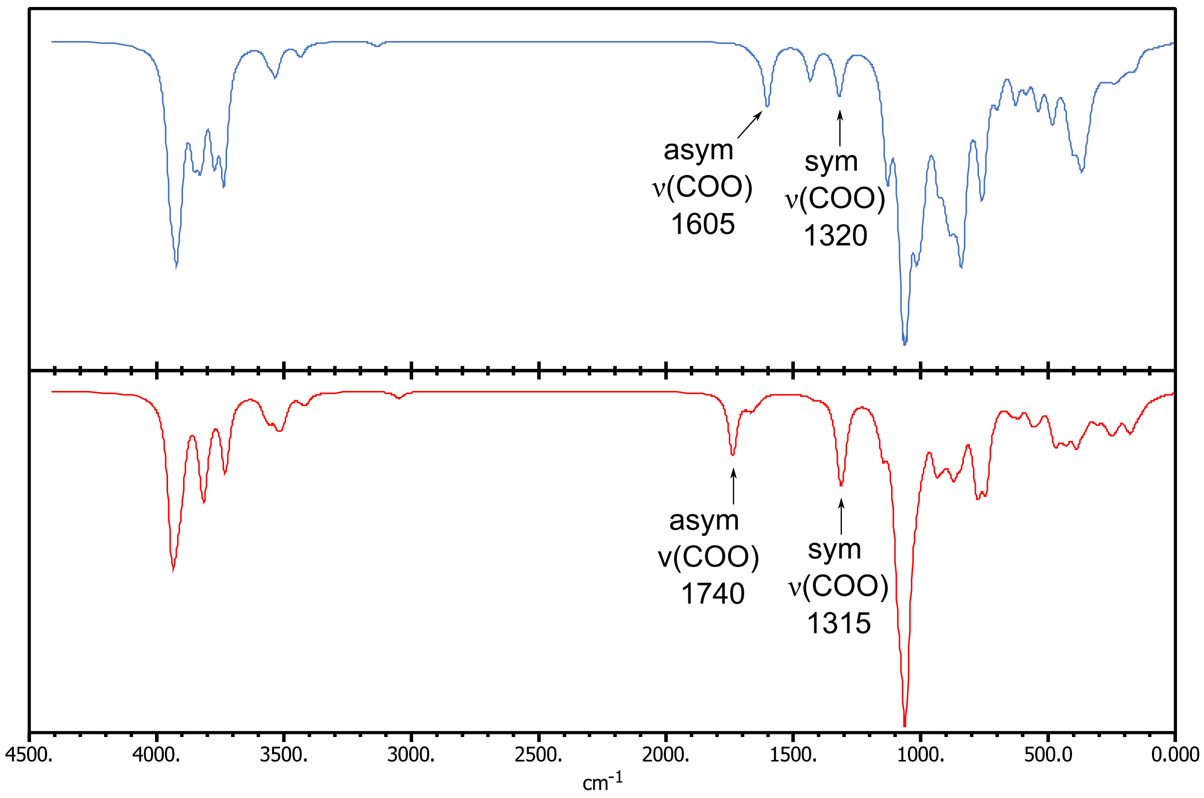

Fig. S22. Simulated IR spectra of zeolite-bound Ru-formates: $[(HOSi(OSi(OH)_3)_3)Ru(OOCH)(NH_3)_n]^+$

Simulated IR spectra of bidentate Ru-formate $[(HOSi(OSi(OH)_3)_3)Ru(OOCH)(NH_3)]^+$ (top), and monodentate Ru-formate: $[(HOSi(OSi(OH)_3)_3)Ru(OOCH)(NH_3)_2]^+$ (bottom), with annotations showing the peaks arising from the symmetric and antisymmetric formate stretches.

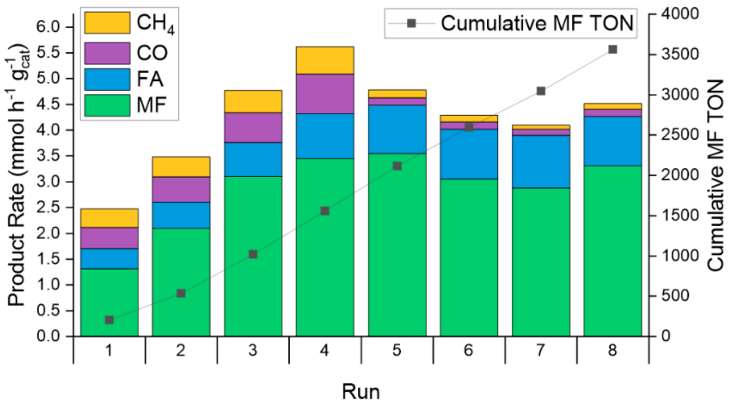

Fig. S23. Recycling: Full product breakdown

Productivity of Ru@Y-1 catalyzed methanol hydrocarboxylation (left axis) and cumulative MF TON for eight reaction cycles. Conditions for each run: Ru@Y-1 (12.5 mg), N-^{n}Pr-Im (250 mg), MeOH (20 mL), CO_2 (20 bar), H_2 (80 bar), 160°C, 16 h. Average of two runs.

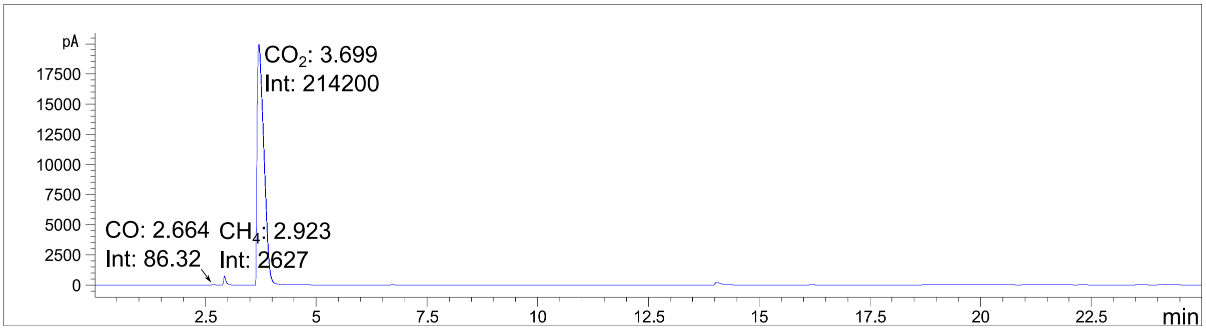

Fig. S24. Representative GC-FID Trace

Representative GC-FID trace, showing the peaks of CO (2.6 min), CH_4 (2.9 min) and CO_2 (3.7 min), and their relative integrations.

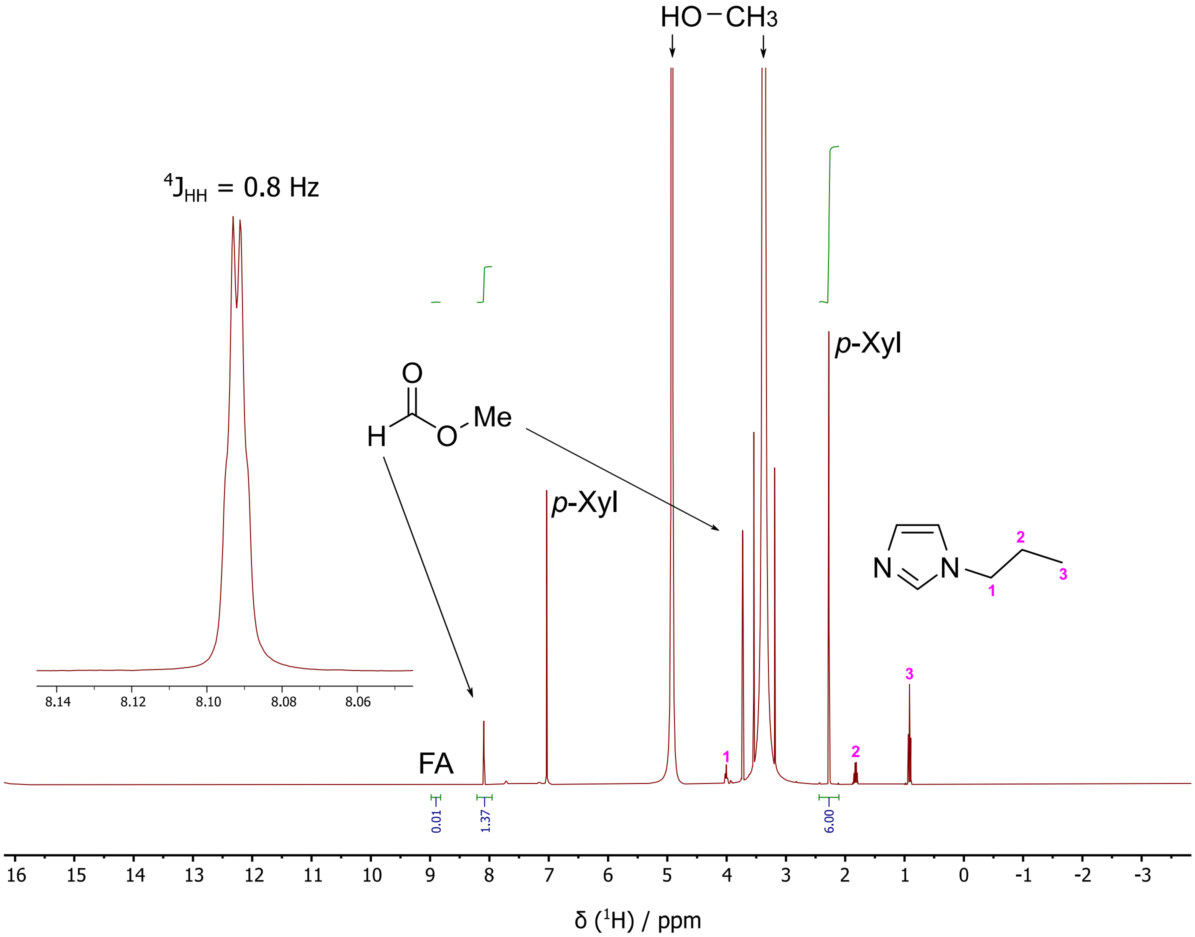

Fig. S25. Representative NMR Spectrum

Representative ^1H NMR Spectrum from reaction mixture after methanol hydrocarboxylation, with integrations of formic acid and methyl formate relative to *para*-xylene CH_3 protons, and inset showing the distinctive quartet of the methyl formate C-*H* proton. Aromatic imidazole protons are visible upon magnification, between 7 and 8.5 ppm. Conditions: Ru@Y-1 (25 mg), N-^{n}Pr-Im (500 mg), MeOH (40 mL), CO_2 (20 bar), H_2 (80 bar), 160℃, 16 h.

Reference	Catalyst Description	Temper-ature / ℃	Time / h	CO_2 pressure / bar	H_2 pressure / bar	TOF[a] / h^{-1}	Activity / mmol h^{-1} g_{cat}^{-1}	MeOH / mL	Base	Selectivity / %	CO_2 Conversion / %	Reaction type
(*23*)	1%Pd-Cu/ZnO/alumina	150	25	10	50[b]	14.6[c] (38.15)[d]	1.0	10	-	96.8[e]	17.6[e]	Batch
(*24*)	Cu/ZnO/alumina	150	25	140	20	43.6[c],[d]	2.4	10	0.07 M Et_3N	>96	2.7[f]	Batch
(*25*)	Au/ZrO_2	120	3	80	80	102	4.1	1.26	-	>99.9	3.9[f],[g]	Batch
(*26*)	Ag/SiO_2	280	-	133	133	n/a	-	-	-	>99.9	1	Flow
(*28*)	Ag/ZrO_2	180	-	133	133	29.1	2.7	-	-	>99.9	1.9	Flow
(*31*)	$RuCl_2\{PMe(CH_2)_2SiO_3\}_3$	100	15	130	85	115	18.1	30	0.6 M Et_3N	100	1.0[f]	Batch
(*29*)	Ru@pDPPE	160	2	20	60	1021 (1426)[h]	100	5.5	0.6 M Et_3N	71.6[j]	4.7	Batch
(*30*)	Ru/N-Me-3-bpp-POP	160	4	20	60	549 (1019)[h]	59.3	37.2	1.5 M Et_3N	53.9[j]	10.0	Batch
This work	Ru@Y-1	160	16	30	80	179 ± 6	18.3 ± 0.6	40	0.11 M N-^{n}Pr-Im	94	18.6 ± 0.5	Batch

[a] Calculated based on all noble metal atoms, unless otherwise stated. [b] 100 bar N_2 as diluent. [c] Calculated from surface Cu sites. [d] Evaluated as initial rate. [e] Without Pd, 90 bar H_2. [f] Calculated using NIST database. [g] After 9 hours. [h] All formates. [j] Liquid products only.

Table S1. Reported methanol hydrocarboxylation catalysts (for comparison)

Reported catalysts for methanol hydrocarboxylation to methyl formate.

**Catalyst**	**Precursor**	**Reductant**	**Calcination Temperature / ℃**	**Support**	**Weight Loading / wt.%**
$[Ru(NH_3)_5(N_2)]^{2+}$@Y-1-RT	$RuCl_3.xH_2O$	$N_2H_4.H_2O$	25	NaY	1[a]
$[Ru(NH_3)_5(N_2)]^{2+}$@Y-1-70	$RuCl_3.xH_2O$	$N_2H_4.H_2O$	70	NaY	1[a]
Ru@Y-1	$RuCl_3.xH_2O$	$N_2H_4.H_2O$	180	NaY	1.0[b]
Ru@Y-0.1	$RuCl_3.xH_2O$	$N_2H_4.H_2O$	180	NaY	0.1[b]
Ru@Y-2	$RuCl_3.xH_2O$	$N_2H_4.H_2O$	180	NaY	2.3[b]
Ru@Y-4	$RuCl_3.xH_2O$	$N_2H_4.H_2O$	180	NaY	4.2[b]
Ru@Y-1-NO_3	$Ru(NO)(NO_3)_3$	$N_2H_4.H_2O$	180	NaY	1.2[b]
Ru@Y-4-NO_3	$Ru(NO)(NO_3)_3$	$N_2H_4.H_2O$	180	NaY	4.4[b]
RuRed@Y-1-RT	$[Ru(NH_3)_5ORu(NH_3)_4ORu\text{-}(NH_3)_5]Cl_6$; RuRed chloride	None	25	NaY	1[a]
RuRed@Y-1	$[Ru(NH_3)_5ORu(NH_3)_4ORu\text{-}(NH_3)_5]Cl_6$; RuRed chloride	None	180	NaY	1[a]
Ru@3ÅMS-0.1	$RuCl_3.xH_2O$	$N_2H_4.H_2O$	180	3Å MS	0.1[b]
Ru@ZSM-5-0.5	$RuCl_3.xH_2O$	$N_2H_4.H_2O$	180	ZSM-5	0.5[b]

[a] Calculated based on initial Ru:NaY ratio. [b] Calculated from ICP-MS.

Table S2. Catalyst Definitions

Definitions and preparation conditions for all supported catalysts produced.

Element	Mass Fraction (%)	Error (%)	Mass Fraction from ICP-MS (%)
O	51.8	3.2	-
Na	4.4	0.9	-
Al	11.8	2.2	-
Si	29.7	4.1	-
Ru	2.3	0.3	2.3

Table S3. EDX quantification

EDX quantification of the chemical species in a particle of Ru@Y-2.

Path	Coordination number	R (Å)	σ^2 (Å^2)	E_0 (eV)	S_0^2	F (%)
**Ru–NH$_3$**	5	2.04 ± 0.01	0.0039 ± 0.0005	22144.7 ± 1.9	0.8	3.81
**Ru–N$_2$**	1	1.83 ± 0.03	0.006 ± 0.003			
**Ru–N–N (MS)[a]**	4	3.00 ± 0.05	0.027 ± 0.010			

[a] Multiple scattering

Table S4. EXAFS fitting parameters for [Ru(NH$_3$)$_5$N$_2$]I$_2$

The Fourier transform was performed with Kaiser-Bessel window applied between $2 < k < 14.5$.

Path	Coordination number	R (Å)	σ^2 (Å^2)	E_0 (eV)	S_0^2	F (%)
**Ru–N/O**	6	2.06 ± 0.01	0.0017 ± 0.00068	22121.4 ± 3.2	0.8	2.73
**Ru–Si1**	1	2.34 ± 0.04	0.0026 ± 0.0026			
**Ru–Si2**	3	3.19 ± 0.02	0.00056 ± 0.00098			
**Ru–Si–O (MS)[a]**	6	3.77 ± 0.03	0.0013 ± 0.0034			
**Ru–O**	12	3.81 ± 0.05	0.0022 ± 0.0055			

[a] Multiple scattering.

Table S5. EXAFS fitting parameters for Ru@Y-1.

The Fourier transform was performed with Kaiser-Bessel window applied between 2 < k < 12. A model generated from putative binding of the Ru centre to the [6,4] vertex of Zeolite Y was used as the input to the FEFF calculation. While the first shell could be modelled with reasonable fidelity, care should be taken interpreting the second shell (ca. R > 2.0) modelling. The Si–O multiple scatter has been modelled with a coordination number of 6 which is almost certainly unphysical – this is to encapsulate the multitude of other multiple scattering paths at a similar distance, instead of modelling each multiple scattering path individually. Similarly, the Ru–O single scatterer found at 3.81 Å and the coordination number of 12 is unphysical – the observed is likely a combination of zeolite O atoms, residual water within pores, and other single- and multiple-scattering paths. Regardless, the second shell appears to be remarkably consistent with the proposed mode of binding.

MF Initial Concentration / M	Temperature / ℃	MF Final Concentration / M
0.20	160	0.18
0.20	180	0.14

Table S6. Methyl formate decarbonylation tests

MF concentrations before and after decarbonylation tests
